# *Sambucus ebulus* L. Fruits: Phytochemistry, Molecular Mechanisms, and Biological Activities in Inflammation, Infection, and Cancer

**DOI:** 10.3390/foods15122106

**Published:** 2026-06-11

**Authors:** Momchil Barbolov, Stoyan Stoyanov, Mladena Radeva, Petyo Boshnakov, Galina Yaneva, Diana Ivanova, Oskan Tasinov

**Affiliations:** 1 Department of Biochemistry, Molecular Medicine and Nutrigenomics, Faculty of Pharmacy, Medical University “Prof. Dr. Paraskev Stoyanov”, 9000 Varna, Bulgaria; momchil.barbolov@mu-varna.bg (M.B.);; 2 Department of Biology, Faculty of Pharmacy, Medical University “Prof. Dr. Paraskev Stoyanov”, 9000 Varna, Bulgaria; stoyan.stoyanov@mu-varna.bg (S.S.);; 3 Department of Ophthalmology and Visual Sciences, Medical University “Prof. Dr. Paraskev Stoyanov”, 9000 Varna, Bulgaria; mladena.radeva@mu-varna.bg; 4 Department of International Economic Relations, University of Economics—Varna, 9000 Varna, Bulgaria; pboshnakov@ue-varna.bg

**Keywords:** *Sambucus ebulus* fruit, dwarf elderberries, phytochemicals, anti-inflammatory, antioxidant, anticancer, antimicrobial, NF-κB, Nrf2

## Abstract

*Sambucus ebulus* L. (dwarf elder) is a polyphenol-rich medicinal plant with a long history of ethnopharmacological use whose biological potential remains substantially underexplored. This narrative review examines the anti-inflammatory, antimicrobial, and anti-proliferative properties of *S. ebulus* fruit preparations and their molecular mechanisms. Literature was retrieved from PubMed, Scopus, and Web of Science (no lower date limit; upper limit May 2026) using “*Sambucus ebulus*” and related terms combined with relevant biological and pathway keywords; studies restricted to non-fruit tissues or lacking phytochemical characterization were excluded or flagged. The fruits contain anthocyanins, flavonols, phenolic acids, proanthocyanidins, and stilbenes that collectively modulate NF-κB, MAPK, JAK/STAT, PI3K/Akt, and Nrf2 signaling. Available evidence is predominantly in vitro, with limited in vivo data, and two human intervention studies. Data support anti-inflammatory, antimicrobial, and anti-proliferative activities that appear to arise from the combined action of multiple phytochemicals. Critical limitations of available research include the absence of clinical trials, limited pharmacokinetic data, lack of standardized preparations, and insufficient formal safety characterization, all of which must be addressed before translational application can be considered.

## 1. Introduction

Medicinal plants have long served as sources of bioactive compounds with therapeutic potential, and their scientific relevance has grown considerably alongside interest in multi-target strategies for complex diseases [1] and an expanding market for plant-derived supplements. Contemporary research increasingly recognizes that chronic conditions such as cancer, metabolic disorders, and infectious diseases share common molecular underpinnings—particularly chronic inflammation and oxidative stress [2]—governed by overlapping signaling networks including NF-κB, MAPKs, JAK/STAT, and the redox sensor Nrf2 [3,4,5]. Phytochemicals that engage several of these pathways simultaneously have therefore attracted attention as prospective therapeutic or adjunct agents.

Within this landscape, *Sambucus ebulus* L. (dwarf elder, danewort) has emerged as a species of growing scientific interest. Widely distributed across Europe and Western Asia, it has a long history of use in traditional medicine across the Balkans, the Middle East, and parts of Asia for the treatment of inflammatory conditions, infections, and pain. Despite this ethnopharmacological relevance, *S. ebulus* remains significantly less studied than its close relative *Sambucus nigra* L. (black elderberry), which has been extensively investigated and commercialized, particularly for antiviral properties [6,7]. This disparity is partly attributable to toxicity concerns related to non-fruit tissues—including the presence of ribosome-inactivating proteins [8]—and to the lack of standardized preparations. The imbalance in research attention is evident through searching PubMed for “*Sambucus ebulus*”, which returns only 43 results over the past ten years (2016–2026), compared with 445 results for “*Sambucus nigra*” over the same period.

Recent investigations have increasingly focused on the fruits of the plant, which have a rich polyphenolic composition dominated by anthocyanins, flavonols (quercetin and kaempferol derivatives), phenolic acids (chlorogenic and caffeic acids), proanthocyanidins, and stilbenes including resveratrol [9]. These compound classes are well recognized for antioxidant, anti-inflammatory, and anti-oncogenic activities mediated through modulation of redox homeostasis and immune signaling [10,11]. Anthocyanins and flavonols in particular suppress NF-κB activation, downregulate pro-inflammatory cytokines (TNF-α, IL-1β, IL-6), and influence MAPK and PI3K/Akt cascades, while simultaneously activating the cytoprotective Nrf2 pathway [12,13]—a profile consistent with multi-target modulation across the inflammation–cancer axis.

*S. ebulus* fruits have also demonstrated antimicrobial activity against viruses [14], bacteria [15], certain fungi [8], and parasites [16], through mechanisms including interference with viral attachment and entry, disruption of microbial membranes, and modulation of host defence responses [17,18]. These effects appear to arise from synergistic interactions among multiple phytochemical constituents rather than single compounds. In parallel, emerging in vitro evidence points to anticancer activity including apoptosis induction, inhibition of proliferation, and suppression of metastatic signaling—mechanistically linked to modulation of Bax/Bcl-2, caspase activation, and inhibition of STAT3 and COX-2, as well as ER stress regulation [19,20]. Direct in vivo validation remains limited.

Several critical limitations currently constrain the translational potential of these findings. Variability in extraction methodology (aqueous, hydroalcoholic, methanolic) produces substantial differences in phytochemical composition and biological activity across studies. Pharmacokinetic data are scarce, and the oral bioavailability of key compounds—particularly anthocyanins—is known to be limited [21]. Well-designed in vivo studies and clinical trials are absent, especially in oncological contexts. All identified current limitations are expanded upon in Section 7 .

Previous reviews have addressed related topics in the *Sambucus* genus over the past decade. Jabbari et al. (2017) provided an early overview of the biological effects and clinical applications of *S. ebulus* across multiple tissues [22], while Merecz-Sadowska et al. (2024) recently reviewed the neuroprotective potential of the *Sambucus* genus—primarily *S. nigra*—as a functional food ingredient, with emphasis on antioxidant and anti-inflammatory mechanisms in the context of brain health and cognitive function [23]. Ebadi (2025) additionally reviewed emerging phytochemical insights and medicinal applications of *S. ebulus* broadly across plant tissues [24]. The present narrative review differs from these existing works in several key aspects. First, it focuses exclusively on the fruits of *S. ebulus*—the safest and most food-relevant tissue—rather than the genus broadly or other tissues. Second, it specifically addresses the inflammation–infection–cancer axis with mechanistic depth, integrating NF-κB, MAPK, JAK/STAT, PI3K/Akt, Nrf2, ER stress, and extracellular vesicle biology in a single framework not previously synthesized for this species. Third, it incorporates the most recent direct *S. ebulus* fruit-specific evidence, including the first human clinical trial data [10], the first colitis-associated cancer in vivo model [19], and the first systematic analysis of ER stress modulation by *S. ebulus* fruit polyphenols [25]. Those findings either post-date or fall outside the scope of prior reviews of the species. Finally, we include discussion of several gaps and limitations in the literature regarding the medicinal potential of *S. ebulus* fruits as a functional food. This narrative review provides a critical outline of the phytochemical composition of *S. ebulus* fruits, the molecular mechanisms underlying their biological activities, and the main translational gaps that must be addressed before their potential as a functional food or nutraceutical can be meaningfully evaluated.

## 2. Literature Search and Review Methodology

This narrative review was conducted following searches of PubMed, Scopus, and Web of Science, with no lower date limit and an upper search date of May 2026. The primary search terms were “*Sambucus ebulus*”, “dwarf elder”, and “dwarf elderberry”, combined using Boolean operators with “anti-inflammatory”, “antioxidant”, “antimicrobial”, “anticancer”, “anti-proliferative”, “apoptosis”, “NF-κB”, “Nrf2”, “MAPK”, “JAK/STAT”, “PI3K/Akt”, “phytochemicals”, “polyphenols”, “anthocyanins”, “phenolic acids”, and “bioavailability”. Reference lists of identified articles were additionally screened for relevant sources not captured by the database searches.

The initial search returned over 340 records. After removal of duplicates and screening of titles and abstracts, about 180 English language articles were assessed for eligibility. Studies were included if they concerned: (1) preparations derived from *S. ebulus* fruits or their constituent phytochemicals evaluated in relevant biological models; (2) original experimental data or substantive reviews of the topic; or (3) mechanistic evidence for signaling pathway modulation by identified phytochemical classes in any experimental system. Studies were excluded if they concerned exclusively non-fruit *S. ebulus* tissues without fruit-specific data, or evaluated nanoparticles without isolating phytochemical contribution. Following full-text review, 121 references were included in the final manuscript. Of these, 28 concern *S. ebulus* directly, of which only 20 are specific to fruit-derived preparations. The remainder concern non-fruit tissues, nanoparticles, or multi-tissue reviews, retained where they provide essential context for the toxicological or novelty discussion. The remaining references support mechanistic inference from constituent phytochemical literature and are clearly flagged as such throughout the text.

As the total *S. ebulus* fruit-specific evidence base is limited, it was not possible to restrict mechanistic discussion to-specific evidence throughout. Potential publication bias toward positive findings is acknowledged as a limitation of both the broader polyphenol literature and the small *S. ebulus*-specific evidence base; null findings are reported where identified. Evidence is categorized throughout by model type—in vitro, in vivo, and human intervention studies—in ascending order of translational relevance. Formal evidence grading (e.g., GRADE) was not applied given the heterogeneity of study designs, extract preparations, and experimental endpoints.

## 3. Phytochemical Composition of *Sambucus ebulus* L. Fruits

The biochemical activity of the fruits reflects their complex polyphenolic composition. Like other *Sambucus* species, the fruits are rich in anthocyanins, flavonols, phenolic acids, proanthocyanidins, and smaller quantities of stilbenes and volatile constituents [9]. Qualitative similarities to *S. nigra* notwithstanding, quantitative differences in phytochemical abundance and antioxidant potential between the two species suggest distinct biological properties [26]. Phytochemical profiles are further shaped by ripening stage, geographical origin, environmental conditions, extraction solvent, and post-harvest processing. Table 1 lists all major polyphenolic and other compounds detected in various extraction protocols for *S. ebulus* fruits.

Anthocyanins are the dominant bioactive class and are responsible for the characteristic dark purple-black coloration of ripe berries. Cyanidin derivatives predominate, with cyanidin-3-glucoside, cyanidin-3-sambubioside, cyanidin-3-sambubioside-5-glucoside, and cyanidin-3-rutinoside among the most consistently identified compounds [26]. Anthocyanin-rich fractions from fruit preparations show strong radical scavenging capacity in chemical antioxidant assays, and mechanistic evidence from related polyphenol-rich systems points to their involvement in modulation of NF-κB, MAPK, and Nrf2 signaling [33,34]. A frequently overlooked consideration is the chemical instability of anthocyanins under common processing and storage conditions. Anthocyanins degrade rapidly in response to elevated temperature, light, oxygen, enzymatic activity, and pH above approximately 3, equilibrating to colorless carbinol pseudobase and chalcone forms with substantially reduced biological activity [35]. Thermal processing steps relevant to typical *S. ebulus* preparations like boiling during infusion, drying of raw material, and ambient storage can reduce anthocyanin content by 30–80% depending on conditions [35,36], with degradation products such as protocatechuic acid and phloroglucinaldehyde exhibiting weaker activity than the parent compounds [36]. The anthocyanin content of a hot water infusion may therefore be substantially lower than that of a cold aqueous extract prepared under controlled laboratory conditions, meaning that anthocyanin-dependent effects demonstrated in vitro may overestimate what is achievable through traditional preparation methods. Standardization of infusion temperature, duration, and storage conditions should therefore be treated as a determinant of biological activity rather than a purely methodological consideration.

Proanthocyanidins (condensed tannins) contribute to both antioxidant and antimicrobial activity through their capacity to interact with proteins, lipid membranes, and metal ions. Their precise composition remains insufficiently characterized relative to anthocyanins, but their presence in crude extracts likely contributes to the broad-spectrum anti-inflammatory and antimicrobial effects observed experimentally [37,38].

Flavonols—principally quercetin, kaempferol, and isorhamnetin derivatives, predominantly as glycosides represent another well-characterized polyphenol class in *S. ebulus* fruits. Quercetin is among the best studied plant flavonoids, with established anti-inflammatory, antioxidant, antimicrobial, and anti-proliferative effects mediated through NF-κB, PI3K/Akt, and JAK/STAT signaling [39,40]. Kaempferol and isorhamnetin derivatives similarly exhibit cytoprotective and anti-inflammatory properties [41,42]. Coexistence of anthocyanins and flavonols within the same extract may enhance overall bioactivity through additive or synergistic interactions [43,44].

Phenolic and organic acids identified include chlorogenic acid, caffeic acid, ferulic acid, quinic acid, and related hydroxycinnamic derivatives. Chlorogenic acid is among the most abundant non-flavonoid phenolics and has been associated with antioxidant, anti-inflammatory, antidiabetic, and antimicrobial effects [45]. Caffeic and ferulic acids contribute additional radical scavenging and membrane-protective activity, partly through Nrf2 pathway activation [46,47], while quinic acid has exhibited direct anti-inflammatory effects and indirect activity through gut microbiome modulation [48,49].

Stilbenes, particularly resveratrol and related derivatives, are present at lower concentrations than anthocyanins or phenolic acids but are of considerable pharmacological interest given their established anti-inflammatory, cardioprotective, and anti-tumorigenic activities [50,51]. Resveratrol interferes with NF-κB activation, cytokine production, oxidative stress responses, and apoptotic signaling, and its presence likely contributes to the broader immunomodulatory and anticancer effects attributed to the plant.

Beyond polyphenols, dwarf elderberries contain sugars, amino acids, vitamins, minerals, fatty acids, and volatile compounds including alcohols, aldehydes, esters, and terpenoids. Although less frequently emphasized, these constituents may affect intestinal absorption, membrane permeability, compound stability, and interactions within the phytochemical matrix—a reminder that biological activity of botanical extracts often cannot be reduced to isolated major compounds alone [9].

Ripening stage is among the main sources of variability across studies. Unripe fruits contain lower anthocyanin concentrations and higher proportions of potentially toxic constituents, while ripening drives substantial anthocyanin biosynthesis and increased antioxidant capacity [9,26]. Geographical origin and growing conditions—soil composition, altitude, climate, and sun exposure—similarly influence polyphenol accumulation, and the marked differences in total phenolic content between studies conducted in Bulgaria, Iran, and Turkey [9,26,28] are consistent with cultivar- and environment-dependent variation, though systematic comparative data across *S. ebulus* populations remain scarce. Extraction methodology represents a further major source of variability: aqueous infusions selectively enrich anthocyanins, hydroxycinnamic acids, and polar proanthocyanidins; hydroalcoholic and methanolic solvents additionally recover flavonol glycosides and stilbenes; and ethyl acetate fractionation concentrates less polar phenolics such as quercetin aglycone and kaempferol derivatives while excluding anthocyanins almost entirely [9,26,28,31]. Direct comparison between studies showed that aqueous-ethanolic preparations at 40–60% ethanol yield higher total polyphenols than purely aqueous extracts, while anthocyanin recovery is optimal at 20–40% ethanol and declines at higher concentrations [27]. Since these compositional differences translate directly into differences in biological activity, results from studies using different extraction protocols cannot be meaningfully compared without parallel phytochemical characterization.

This review focuses specifically on ripe fruit-derived preparations. Ribosome-inactivating proteins (RIPs), lectins, and cyanogenic glycosides—predominantly associated with leaves, rhizomes, stems, and unripe tissues—are responsible for the toxicological concerns surrounding *S. ebulus* [52] but are not considered major constituents of properly processed ripe fruit preparations and fall outside the scope of the present discussion.

Taken together, the phytochemical composition of the fruits provides a plausible biochemical basis for their reported biological activities. Rather than acting through a single dominant molecule, the observed effects of the fruits could arise from the coordinated activity of multiple phytochemical classes modulating interconnected signaling pathways in inflammation, oxidative stress, and cellular survival—the mechanisms of which are addressed in the following sections.

## 4. Immunomodulatory Effects of *Sambucus ebulus* L. Fruits

Chronic inflammation is now well established as a shared mechanistic driver across a wide range of pathological conditions such as cardiovascular disease, metabolic disorders, neurodegeneration, autoimmune disease, and cancer by promoting tissue remodeling, oxidative stress, and genomic instability through sustained dysregulation of immune signaling [2,53]. Plant-derived polyphenols capable of modulating signaling pathways underpinning this process have attracted growing interest as therapeutic or chemopreventive agents [54]. *S. ebulus* fruits are a particularly relevant source in this regard: their high content of anthocyanins, flavonols, and phenolic acids consistently confers antioxidant and anti-inflammatory activity in experimental models, with evidence pointing to both individual compound effects and synergistic interactions within the intact phytochemical matrix [9,26]. A summary of direct experimental evidence for immunomodulatory and anti-inflammatory activity from *S. ebulus* fruit extract studies is presented in Table 2.

### 4.1. NF-κB Signaling Pathway

Among the molecular targets implicated in anti-inflammatory activity, NF-κB occupies a central role as a master regulator of cytokine, chemokine, adhesion molecule, and inflammatory enzyme transcription. Under basal conditions it is held inactive in the cytosol by IκBα; pro-inflammatory stimuli such as LPS, ROS, and cytokines activate the IKK complex, triggering IκBα degradation and NF-κB nuclear translocation [56].

Constituent phytochemicals of *S. ebulus* fruits—principally anthocyanins, quercetin derivatives, chlorogenic acid, and resveratrol—are known from other experimental systems to interfere with NF-κB activation at several levels, including inhibition of IκBα phosphorylation, suppression of p65 nuclear translocation, and reduced transcriptional activation of downstream inflammatory genes [57]. Direct evidence specifically for *S. ebulus* fruit extracts is more limited but meaningful: in LPS-stimulated J774A.1 macrophages, aqueous fruit extract suppressed transcription of IL-1β, IL-6, TNF-α, COX-2, iNOS, and ICAM-1, with iNOS protein-level reduction confirmed by Western blot and effects comparable to salicylic acid [9]. A human intervention study in 53 healthy volunteers consuming *S. ebulus* fruit infusion for four weeks reported significant decreases in serum IL-6, TNF-α, and IL-8 [10]. This study does however have its limitations: it lacked a placebo control, evaluated only a single dose without dose–response analysis, used a non-batch-standardized infusion, and was conducted in healthy volunteers rather than patients with inflammatory disease. This constrains the conclusions that can be drawn, though the study remains the most clinically relevant evidence available for *S. ebulus* fruit preparations. These, alongside the polyposis study cited later, are the available direct evidence for NF-κB pathway suppression by *S. ebulus* fruit preparations specifically; the broader mechanistic detail—including IKK inhibition and nuclear translocation assays—has not been shown for *S. ebulus* extracts and is inferred from constituent phytochemical literature.

Of the cited *S. ebulus* studies, only Tasinov et al. [9] performed direct protein-level analysis (Western blot for iNOS, p-eIF2α, ATF6α, and CHOP); nuclear translocation assays for p65 or Nrf2 have not been performed, and broader pathway-level mechanistic descriptions are inferred from constituent phytochemical literature.

### 4.2. MAPK Signaling Pathways

MAPK signaling is another key target. The family comprises three principal branches—ERK1/2, JNKs, and p38. They collectively regulate inflammatory gene expression and cytokine production downstream of Toll-like receptors, cytokine receptors, and oxidative stress [58]. ERK and JNK converge on activator protein-1 (AP-1), while p38 additionally activates ATF-2 and MSK1, the latter directly enhancing NF-κB-driven transcription and providing a point of crosstalk between the two pathways [58].

Quercetin and anthocyanin derivatives present in *S. ebulus* fruits suppress phosphorylation of ERK, JNK, and p38 in inflammatory models, reducing AP-1 activation and downstream expression of COX-2, IL-6, and matrix metalloproteinases [57,59,60]. This suppression is typically partial rather than complete, which may be favorable given that MAPK branches also mediate tissue repair and adaptive stress responses. As with the other pathways discussed here, these effects are documented for isolated constituents in other systems and have not been directly demonstrated for *S. ebulus* fruit extracts.

### 4.3. Regulation of Pro-Inflammatory Cytokines and Inflammatory Enzymes

A consistent finding across studies involving dwarf elderberry extracts is the downregulation of major pro-inflammatory cytokines, particularly TNF-α, IL-1β, and IL-6 [9,10]. These cytokines drive leukocyte recruitment, endothelial activation, and chronic tissue damage, which if sustained contributes to autoimmune disease, chronic infection, and tumorigenesis.

The observed suppression of cytokine production by *S. ebulus* preparations is likely mediated through combined inhibition of NF-κB and MAPK signaling pathways. Of constituent phytochemicals, anthocyanins and quercetin derivatives are especially well characterized in this regard [34,57]. Additionally, reduced expression of COX-2 and iNOS has been observed in LPS-stimulated macrophage models treated with *S. ebulus* fruit extract, with confirmed protein-level reductions in iNOS [9]. In a model of chronic eosinophilic inflammation, Pourgholamali et al. demonstrated that hydroalcoholic *S. ebulus* fruit extract (50–1000 µg/mL) significantly reduced GM-CSF levels in human nasal polyp tissue ex vivo, and simultaneously increased apoptosis and pro-apoptotic Bax/Bad gene expression in eosinophilic inflammatory cells. This suggests that *S. ebulus* fruit preparations may attenuate chronic inflammatory cell survival through combined suppression of cytokine signaling and promotion of apoptosis [55]. Chlorogenic acid, quercetin, and resveratrol are known to individually inhibit iNOS transcription and modulate cytokine balance [61,62,63].

### 4.4. JAK/STAT Signaling Axis

The JAK/STAT pathway transduces cytokine and growth factor signals directly to the nucleus and may also be targeted by dwarf elderberries. Cytokine binding activates receptor-associated JAK kinases, which phosphorylate STAT proteins—principally STAT3—that dimerize and translocate to the nucleus to drive transcription of genes governing proliferation, survival, and immune regulation. Persistent STAT3 activation in particular links chronic inflammation to cancer progression, promoting expression of IL-6, Bcl-2, survivin, cyclin D1, and VEGF [64].

Quercetin and anthocyanins such as cyanidin-3-O-glucoside present in *S. ebulus* fruits suppress STAT3 phosphorylation and nuclear activity in various experimental systems [65,66,67]. Because STAT3 and NF-κB share an IL-6-driven feed-forward loop—NF-κB induces IL-6, which activates STAT3, which in turn sustains NF-κB activity—simultaneous modulation of both may produce amplified anti-inflammatory and anti-tumorigenic effects. As with the other pathways, direct mechanistic studies of JAK/STAT signaling in response to *S. ebulus* extracts remain scarce, and these effects are inferred from constituent phytochemicals in other systems.

### 4.5. PI3K/Akt Pathway

The PI3K/Akt pathway is activated downstream of receptor tyrosine kinases by growth factors, cytokines, and insulin, generating PIP3 that recruits and activates Akt [68]. Akt promotes survival and proliferation through several effectors—inactivating pro-apoptotic FOXO transcription factors, activating mTORC1, stabilizing cyclin D1 and c-Myc via GSK-3β, and inactivating BAD [68]. In inflammatory settings it also phosphorylates and activates IKK, a feed-forward link to NF-κB directly relevant to the network discussed here [68].

Separate polyphenolic constituents of *S. ebulus* fruits modulate this pathway at several levels, though the direction and magnitude of effect are context-dependent. In cancer cell models characterized by constitutive PI3K/Akt activation and elevated oxidative stress, quercetin inhibits the p110 catalytic subunit of PI3K, reducing PIP3 generation and Akt Ser473 phosphorylation, thereby promoting FOXO nuclear translocation and pro-apoptotic gene expression [69,70]. Resveratrol independently inhibits mTORC1 in an ATP-competitive manner, activating autophagy through ULK1 disinhibition and simultaneously reducing downstream pro-survival signaling [71]. Anthocyanins and kaempferol have been shown in various cell-based models to reduce Akt phosphorylation, shifting the balance toward FOXO-dependent apoptosis and cell cycle arrest [41,65]. In contrast, in non-malignant cell types under metabolic or oxidative stress, moderate PI3K/Akt activation by polyphenols may contribute to cytoprotective responses including Nrf2 stabilization via Akt-mediated Keap1 phosphorylation and to maintenance of endothelial integrity and insulin sensitivity [72]. This duality reflects the known concentration- and context-dependence of polyphenol action—the same compound may inhibit a hyperactivated oncogenic pathway in tumor cells while supporting physiological homeostatic signaling in normal tissue [25].

The crosstalk between PI3K/Akt and other pathways discussed in this review is extensive. The Akt-IKK-NF-κB axis represents one of the most clinically relevant feed-forward connections linking growth factor signaling to inflammatory gene expression [68], and its disruption by quercetin and resveratrol simultaneously at the PI3K and NF-κB levels may produce more durable anti-inflammatory effects than inhibition of either pathway alone. The PI3K/Akt-mTORC1 axis further intersects with the ER stress response: chronic mTORC1 activation promotes ER protein load and UPR activation, while Akt-mediated modulation of eIF2α kinases influences the integrated stress response [25]. Additionally, Akt and ERK1/2 share upstream RTK inputs through RAS, creating compensatory signaling redundancy, which is a well-documented resistance mechanism in cancer, and therefore polyphenols with dual PI3K/Akt and MAPK inhibitory activity, such as quercetin, may be particularly suited to modulate [70,73].

### 4.6. Nrf2 Activation and Redox Regulation

Oxidative stress and inflammation are tightly linked biological processes that amplify one another through ROS-mediated signaling mechanisms. Excessive ROS production activates inflammatory pathways such as NF-κB and MAPKs, which, among other effects, cause immune cells to generate additional ROS through enzymes like NADPH oxidase and myeloperoxidase, perpetuating a self-amplifying cycle of tissue damage [74].

Nrf2 is the principal regulator of cellular antioxidant defenses. Under basal conditions it is held by Keap1 in the cytoplasm and degraded; oxidative or electrophilic stress releases Nrf2 for nuclear translocation and activation of antioxidant response element (ARE)-dependent genes including HO-1, glutathione-related enzymes, SOD, and NQO1 [75].

Nrf2 and NF-κB are linked through extensive crosstalk: competition for transcriptional coactivators such as CBP/p300, NF-κB-mediated HDAC3 recruitment suppressing ARE-driven transcription, and inhibition of inflammatory signaling by Nrf2 targets such as HO-1 [74].

We should distinguish between two levels of evidence in this section. Directly measured antioxidant outcomes for *S. ebulus* fruit preparations include: DPPH and ABTS radical scavenging capacity across multiple extract types [26,27,28]; increased total serum antioxidant capacity and reduced lipid peroxidation markers in healthy human volunteers consuming fruit infusion [11]; and suppression of oxidative stress markers in the AOM/DSS colon cancer mouse model [19]. Nrf2 pathway activation, by contrast, has not been directly proven for *S. ebulus* fruit extracts in any published study—no *S. ebulus* study has measured Nrf2 nuclear translocation, ARE-reporter activity, or Nrf2-dependent gene expression (HO-1, NQO1, SOD) in response to fruit extract treatment. The involvement of Nrf2-mediated mechanisms is therefore inferred from: (a) the known Nrf2-activating properties of the constituent phytochemicals anthocyanins [76], quercetin [77], chlorogenic acid [78], and resveratrol [79] in other experimental systems; and (b) the indirect observation that antioxidant enzyme activity increased in human volunteers consuming the fruit infusion [11]. This is consistent with but does not explicitly confirm Nrf2 activation. This distinction is maintained throughout the section.

The dual role of Nrf2 in cancer biology warrants explicit discussion. While Nrf2 activation is broadly tumor-suppressive in the context of chemoprevention through reduction in oxidative DNA damage, limiting carcinogen bioactivation, and suppression of inflammation-mediated malignant transformation, constitutive Nrf2 activation in established tumors represents a distinct and clinically important problem [80]. Somatic loss-of-function mutations in *KEAP1* and gain-of-function mutations in *NRF2* itself are among the most frequent genetic events in lung, oesophageal, and head and neck cancers, and drive enhanced antioxidant buffering, drug efflux, and metabolic reprogramming that additively give rise to resistance to chemotherapy and radiotherapy [80,81]. The implications for dietary polyphenol-mediated Nrf2 activation in patients with established malignancies are therefore uncertain: moderate, transient activation in normal or pre-malignant tissue may be chemopreventive, but the same activity in tumor cells harboring pre-existing Nrf2 pathway mutations could potentially support rather than suppress tumor survival. This distinction has not been addressed in any *S. ebulus* study and is a consideration for future research, particularly in the context of adjunctive use during oncological treatment.

Collectively, the available evidence indicates that the anti-inflammatory activity of *S. ebulus* fruits arises from simultaneous modulation of multiple interconnected pathways rather than inhibition of a single target. However, most pathway-level detail derives from individual constituent phytochemicals; direct evidence that whole fruit extract engages these pathways concurrently remains limited to the NF-κB and Nrf2 findings discussed above [9,10].

## 5. Antimicrobial Effects of *Sambucus ebulus* L. Fruits

The growing burden of antimicrobial resistance and the emergence of novel viral pathogens have renewed interest in polyphenol-rich plants, which can target multiple microbial processes and modulate host immune responses simultaneously [82,83]. *S. ebulus* fruits have been studied for antiviral, antibacterial, antifungal, and antiparasitic activity, with available evidence suggesting cooperative interactions among phytochemical classes rather than a single dominant compound [15,84]. Direct evidence from *S. ebulus* fruit extract studies is summarized in Table 3.

### 5.1. Antiviral Activity

Antiviral activity has received the most attention among the antimicrobial properties of *S. ebulus*. Ghaffari et al. demonstrated that hydroalcoholic extract of dwarf elderberries inhibited HSV-1 infection in Vero cells, with a significant reduction in HSV-1 antigen expression confirmed by immunofluorescence [14]. Zahmanov et al. profiled flavonoid glycosides from 70% methanol fruit extract and documented anti-HSV-1 activity in purified flavonoid fractions, identifying quercetin-3-rutinoside and isorhamnetin-3-rutinoside as the principal active constituents [29]. A comparative study testing *S. nigra* and *S. ebulus* fruit and leaf extracts against HSV-2 found strong virucidal activity in *S. nigra* preparations; the purified *S. ebulus* berry extract did not have significant activity in this system, likely reflecting differences in anthocyanin and phenolic acid content between the two species [85]. These findings collectively illustrate that antiviral potency in *Sambucus* preparations is tissue- and composition-dependent, and results should not be generalized across extract types without phytochemical characterization.

The mechanistic basis of these effects likely spans multiple stages of the viral life cycle. Among the constituent phytochemicals, quercetin interferes with viral entry and targets viral proteases and polymerases [92,93], resveratrol suppresses replication of several RNA and DNA viruses via NF-κB and redox-sensitive signaling [94,95], and anthocyanins and flavonols alter membrane fluidity and surface charge to limit adsorption of enveloped viruses [96,97]. These phytochemicals may additionally attenuate the excessive inflammatory response accompanying viral infection [9]. These mechanisms are inferred from isolated constituents in other systems; in vivo and mechanistic data specific to *S. ebulus* fruit preparations remain scarce.

### 5.2. Antibacterial Activity

Fruit extracts of dwarf elder have documented antibacterial activity against both Gram-positive and Gram-negative species, though susceptibility varies considerably depending on extract preparation and the target organism. Salehzadeh et al. reported that methanolic *S. ebulus* extract inhibited all 16 clinical MRSA isolates tested, with an MIC of 15 mg/mL against the *S. aureus* ATCC reference strain [15]. Kayıran et al. characterized dried fruit methanol extract and fresh fruit juice by LC-MS/MS and found moderate activity against *E. coli*, *P. mirabilis*, and *S. aureus*, alongside variable susceptibility among yeast species [28]. Rodino et al. evaluated ethanolic fruit extract against a broader panel of both Gram-positive and Gram-negative organisms—including *B. subtilis*, *E. faecalis*, *B. cereus*, *S. aureus*, *P. fluorescens*, and *E. coli*—and reported inhibitory activity against most tested strains, with the strongest results against *P. fluorescens* and *E. faecalis* [86]. Activity correlated with total phenolic and flavonoid content across all three studies, consistent with polyphenols as the primary mediators.

The antibacterial mechanisms of the constituent polyphenols are multifactorial. They include disruption of membrane integrity and leakage of intracellular contents [28,98], inhibition of bacterial DNA gyrase and topoisomerase IV by quercetin derivatives [99], interference with membrane-associated energy metabolism by chlorogenic and caffeic acids [100], impairment of adhesion and biofilm formation by proanthocyanidins [98,101], and metal chelation restricting access to essential trace elements [102]. Synergy with conventional antibiotics has been proposed on the basis of polyphenol mechanisms in other systems [101], but no cited *S. ebulus* study performed formal synergy assays (checkerboard dilutions, FICI), so these interactions remain hypotheses rather than demonstrated properties of dwarf elder preparations.

Gram-positive organisms are generally more susceptible than Gram-negative species, reflecting the protective outer membrane of the latter [98]. Several cited studies use methanolic extracts [15,28,86], which maximize polyphenol recovery but are not food-relevant preparations; their results are best read as proof-of-concept rather than direct support for dietary or nutraceutical use, where aqueous or food-grade preparations would apply. Variability in solvent, fruit maturity, origin, and methodology further limits cross-study comparison.

### 5.3. Antifungal and Antiparasitic Activity

The antifungal and antiparasitic properties of *S. ebulus* fruit extracts are the least well characterized of the antimicrobial domains reviewed here, and the available evidence should be interpreted as preliminary. Regarding antifungal activity, Kayıran et al. found that dried fruit methanol extract displayed activity against *C. tropicalis* (MIC 312.5 mg/L), while *C. albicans*, *C. parapsilosis*, *S. epidermidis*, and *T. rubrum* were resistant to all preparations tested [28]. Rodino et al. additionally reported inhibition of mycelial growth in *Botrytis cinerea*, *Rhizoctonia solani*, and *Phytophthora infestans* with ethanolic fruit extract [86], though these are plant rather than human pathogens and their clinical relevance is limited. Separate work showed complete inhibition of *Saprolegnia parasitica* hyphal growth at ≥5% *S. ebulus* ethanolic extract concentration [87]. Proposed mechanisms include disruption of fungal membranes and inhibition of adhesion and biofilm formation [87,101], though in vivo validation for *S. ebulus* fruit is lacking.

Antiparasitic activity has also been reported for *S. ebulus* fruit preparations against several protozoan parasites. Rahimi-Esboei et al. demonstrated significant in vitro cytotoxicity against *Giardia lamblia* cysts isolated directly from patients, identifying *S. ebulus* fruit as a candidate natural antigiardial agent [88]. Gholami et al. similarly showed concentration- and time-dependent scolicidal activity against *Echinococcus granulosus* protoscoleces in vitro [89]. Against *Leishmania major*, available evidence points primarily to leaf rather than fruit preparations—Kadkhodamasoum et al. found that fruit extract exhibited significantly weaker antiparasitic activity than leaf extract in vitro [90], and in vivo stimulation of cellular immune responses was likewise attributed to the leaf fraction [16]. Activity against *Toxoplasma gondii* has been reported for silver nanoparticles synthesized using *S. ebulus* fruit extract both in vitro and in vivo [91], although the contribution of the nanoparticles themselves versus the phytochemical coating cannot be distinguished, and these results should not be attributed to the fruit extract phytochemicals alone.

As in other domains, crude extracts frequently outperform isolated compounds at equivalent concentrations, pointing to additive or synergistic interactions among phytochemical classes [9,28]. This multi-target activity, while preliminary, points toward possible adjuvant applications that warrant further investigation.

## 6. Anti-Proliferative and Pro-Apoptotic Potential of *Sambucus ebulus* L. Fruits

Cancer arises through a combination of dysregulated proliferation, apoptosis resistance, chronic inflammation, oxidative stress, immune evasion, and angiogenesis [103]. As these processes are interconnected, there is growing interest in phytochemicals and combinations thereof that can engage multiple hallmarks of cancer simultaneously. *S. ebulus* fruits contain several phytochemicals independently known to influence oncogenic signaling pathways [9], and available in vitro data suggest anti-proliferative, pro-apoptotic, and immunomodulatory activity. It must be emphasized at the outset, however, that clinical evidence for *Sambucus ebulus* preparations is entirely absent, that direct in vivo data are limited to two studies [19,20], and that in vitro concentrations associated with cytotoxic effects are generally far above those achievable in plasma or tissue following oral administration given the well-documented limitations of polyphenol bioavailability [104]. The findings reviewed in this section should therefore be interpreted as exploratory mechanistic evidence rather than as demonstration of anti-cancer efficacy.

### 6.1. Evidence from Cell Line Studies

In vitro studies have reported cytotoxic and anti-proliferative activity of *S. ebulus* fruit extracts against multiple cancer cell lines, including models of hepatocellular carcinoma, colorectal cancer, cervical cancer, lung cancer, and triple-negative breast cancer. Saravi et al. reported that ethyl acetate extract of *S. ebulus* fruits showed lower IC_50_ values in hepatocellular (HepG2) and colorectal (CT26) cancer cell lines compared to non-malignant cell lines (CHO and rat fibroblasts), suggesting some degree of selective cytotoxicity, though potency was lower than etoposide [105]. Anti-proliferative activity has also been detected against A-549 (lung), LS-174T (colorectal), and HeLa (cervical) cell lines, although it was more strongly associated with the leaves and not the fruit [32].

A more mechanistically detailed study by Omrani et al. evaluated petroleum ether *S. ebulus* fruit extract in MDA-MB-231 triple-negative breast cancer cells and in a mouse xenograft model. The *S. ebulus* extract significantly increased expression of Bax, Bak, p53, and c-MYC, with no toxicity observed in normal breast cells (MCF-10A), and the authors concluded it may represent a safe compound for eliminating breast cancer cells [20]. These findings represent one of the few studies combining in vitro and in vivo data for dwarf elder in a cancer context, though the molecular mechanisms underlying malignant cell selectivity were not fully elucidated.

Polyphenol-rich and especially anthocyanin-rich fractions tend to show dose-dependent effects in vitro—cytoprotective at low concentrations, pro-oxidant and pro-apoptotic at higher ones [105,106]. However, the IC_50_ values reported for *S. ebulus* extracts (tens to hundreds of µg/mL) [32,105] far exceed achievable plasma polyphenol concentrations, a limitation examined in detail in Section 7.2. In the absence of pharmacokinetic data for *S. ebulus*, the in vivo relevance of these in vitro concentrations is uncertain.

### 6.2. Evidence from Animal Models

Direct in vivo evidence for dwarf elder in cancer models remains limited. The most informative animal study to date is that of Kaya et al., who used an AOM/DSS colitis-associated colon cancer mouse model to evaluate *S. ebulus* fruit extract (100 mg/kg/day), investigating its modulatory role on oxidative stress, apoptosis, and TRP channel activity in the colon of AOM/DSS mice [19]. This model is relevant because it mimics the well-established inflammation–cancer axis in colorectal carcinogenesis [107]. Beyond this, much of the available animal data comes from abovementioned studies focused on anti-inflammatory or antioxidant endpoints rather than tumor suppression per se; interpretation in an oncological context should therefore be made cautiously. Pharmacokinetic data and dose–response characterization in tumor-bearing animals are essentially lacking.

### 6.3. Apoptosis and Mitochondrial Signaling

Induction of apoptosis is among the most consistently proposed anti-cancer mechanisms of polyphenol-rich *S. ebulus* extracts, although it is mostly an extrapolation of known effects of its individual phytochemical constituents. Several of them—quercetin, resveratrol, and kaempferol—are known to modulate both the intrinsic mitochondrial pathway through downregulation of proliferative signals and, to a lesser extent, extrinsic apoptotic signaling in cancer cells [69,108,109]. Other phytochemicals like anthocyanins and chlorogenic acid have exhibited anti-apoptotic effects on non-malignant cells undergoing ER or oxidative stress [110,111].

Quercetin has been documented to shift the ratio of the process’s main controllers Bax and Bcl-2 in favor of apoptosis, reduce mitochondrial membrane integrity, and activate caspase cascades in multiple cancer cell types [108]. Resveratrol promotes apoptosis and can induce mitochondrial dysfunction across a range of tumor models through PI3K/Akt, STAT3, and MAPK pathway modulation [109]. Consistent with this, the Omrani et al. study found that *S. ebulus* extract upregulated Bax, Bak, and p53 in MDA-MB-231 cells, pointing toward mitochondria-mediated apoptosis as the operative mechanism [20]. Of importance is that Omrani et al. [20] assessed apoptosis exclusively through gene expression analysis of Bax, Bak, p53, and c-MYC; caspase activation assays, mitochondrial membrane potential measurements, and intracellular ROS quantification were not performed. Kaya et al. [19] assessed oxidative stress markers and caspase pathway gene expression in the AOM/DSS model, providing somewhat broader mechanistic coverage, though again without direct protein-level caspase activity or mitochondrial membrane potential data. The interpretation of mitochondria-mediated apoptosis as the operative mechanism is therefore partly inferential, supported by the Bax/Bcl-2 expression data and by analogy with constituent phytochemical studies in other systems.

An additional consideration is the context-dependent pro-oxidant behavior of polyphenols. In cancer cells with elevated baseline ROS due to increased glucose metabolism and compromised antioxidant buffering, polyphenols can tip redox balance toward apoptotic signaling, while acting protectively in normal cells [106]. This selectivity, as documented in vitro for *S. ebulus* fruit extracts [20,105], is mechanistically attractive but requires in vivo validation.

### 6.4. NF-κB, STAT3, and PI3K/Akt in Cancer Contexts

The NF-κB, STAT3, and PI3K/Akt pathways discussed in Section 4 are also central to tumor biology, driving anti-apoptotic protein expression, proliferation, immune evasion, and metastasis [103].

Anthocyanins and quercetin derivatives suppress NF-κB through IKK inhibition and reduced IκBα degradation in several experimental systems [9,40]. Quercetin additionally inhibits Akt activity and downstream mTOR signaling, promoting apoptosis and reducing proliferation in colorectal and other cancer cell lines [70,73]. Resveratrol modulates STAT3 through PI3K/Akt-dependent and independent mechanisms, reducing expression of cyclin D1, survivin, and Bcl-2 [109]. These converging actions on interconnected oncogenic pathways represent a genuine theoretical advantage of polyphenol-rich extracts in cancer: partial simultaneous modulation of multiple nodes may be more durable than complete inhibition of a single target.

### 6.5. Anti-Metastatic and Anti-Angiogenic Effects

No migration or invasion assays, tube formation assays, or VEGF-related functional studies have been directly performed using *S. ebulus* fruit extracts to our knowledge. The following discussion is therefore entirely inferential, based on suppression of NF-κB and STAT3-associated inflammatory gene expression demonstrated in *S. ebulus* macrophage models [9] and known effects of constituent phytochemicals on MMP, VEGF, and EMT markers in other experimental systems, and should be read as a mechanistic hypotheses rather than a documented property of the extract.

Tumor progression depends critically on MMP-mediated matrix degradation, angiogenesis driven by VEGF, and epithelial-to-mesenchymal transition (EMT). Suppression of NF-κB and STAT3 by phytochemicals present in *S. ebulus* fruit may reduce MMP-2/9 and VEGF expression, potentially impairing metastatic dissemination [9,70]. Anti-inflammatory modulation of the tumor microenvironment—reducing recruitment of tumor-associated macrophages and fibroblasts through lower cytokine production—may further indirectly limit tumor-promoting conditions, though this has not been directly investigated for *S. ebulus* fruit extracts specifically.

### 6.6. MicroRNA and Epigenetic Regulation

No studies have directly investigated miRNA expression or extracellular vesicle biology in response to *S. ebulus* fruit extracts. The following discussion is based entirely on evidence from constituent phytochemicals—quercetin, resveratrol, and anthocyanins—in other experimental systems, and should be understood as a mechanistic hypothesis regarding potential mechanisms that would warrant direct investigation in future work.

Modulation of microRNAs (miRNAs) is an emerging dimension of in vitro polyphenol anticancer activity, as miRNAs post-transcriptionally regulate oncogenic and tumor-suppressive networks [112]. Several individual phytochemicals identified in *S. ebulus* fruits can influence miRNA expression relevant to cancer in vitro. Resveratrol and quercetin have both been shown to suppress oncogenic miR-21—whose targets include PTEN, PI3K/Akt, STAT3, and NF-κB pathways—and to upregulate tumor-suppressive members of the miR-34a and let-7 families in various cancer models [112,113,114]. Quercetin additionally modulates miRNAs associated with NF-κB signaling, apoptosis resistance, and metastatic phenotypes [114]. Direct studies investigating miRNA regulation by *S. ebulus* extracts are absent from the literature, but the phytochemical composition of the fruits makes this a reasonable topic for future research.

### 6.7. Limitations and Perspectives

The principal limitation of the current evidence is the near-exclusive reliance on in vitro systems, often using crude extracts at supraphysiological concentrations. The one breast cancer study combining in vitro and in vivo evidence [20] and the AOM/DSS colon cancer model [107] are exceptions, but both are single studies with limited mechanistic depth. Clinical data are absent. Substantial heterogeneity in extraction methodology, phytochemical composition, and experimental endpoints further limits reproducibility and inter-study comparison. The dual redox behavior of polyphenols—protective in normal tissue, potentially pro-oxidant in malignant cells—requires careful dose-optimization and tissue-specific safety evaluation before any translational application is considered [25]. Future work should prioritize standardized extract characterization, well-powered in vivo tumor models, pharmacokinetic profiling, and ultimately clinical pilot studies to determine whether the encouraging in vitro signals from *S. ebulus* research translate to meaningful activity in vivo.

## 7. Discussion

**Figure 1:**
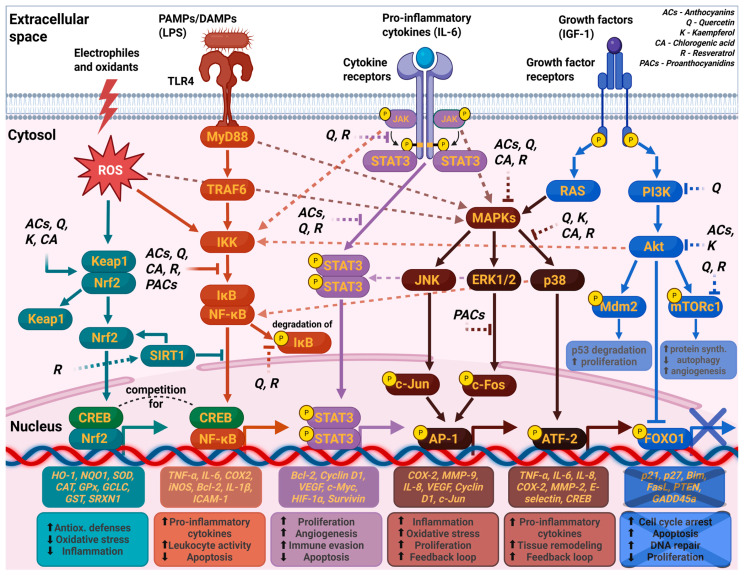
Molecular targets of the most represented *Sambucus ebulus* L. fruit phytochemicals across main inflammatory and oncogenic signaling pathways across experimental models. Anthocyanins (ACs), quercetin (Q), kaempferol (K), chlorogenic acid (CA), resveratrol (R), and proanthocyanidins (PACs) act at multiple nodes across the NF-κB, JAK/STAT3, MAPK, PI3K/Akt, and Nrf2 pathways simultaneously, engaging shared upstream kinases (IKK, MAPKs, PI3K) and convergent transcription factors (NF-κB, STAT3, AP-1, ATF-2) rather than single isolated targets. Extensive crosstalk, including the NF-κB-IL-6-STAT3 feed-forward loop, Akt-mediated IKK activation, and p38/MSK1->NF-κB reinforcement, amplifies inflammatory and tumorigenic programs that these phytochemicals collectively disrupt. Nrf2 activation provides a counter-regulatory axis that simultaneously suppresses ROS-driven inflammatory amplification. The synergistic multi-target engagement of the intact phytochemical matrix likely underlies the broad anti-inflammatory, antimicrobial, anti-proliferative and pro-apoptotic activity reported for *S. ebulus* fruit preparations. Of note is that only the IKK and Nrf2/Keap1 interactions are proven for *S. ebulus* fruit extracts [9,10,11,26] (faded solid lines), while all other phytochemical interactions refer to individual constituent phytochemicals (faded dashed lines), and therefore represent mechanistic hypotheses rather than directly demonstrated effects of fruit preparations. Solid arrows indicate activation; flat-ended arrows indicate inhibition; dashed arrows indicate crosstalk. Some activation steps omitted for clarity. Created in Biorender. Barbolov, M. (2026) https://app.biorender.com/illustrations/6a06f453ab13d3f1a444fbd8?slideId=10240550-2761-4a5d-9629-552df297f26b (accessed on 5 June 2026).

No single *S. ebulus* fruit extract study has simultaneously evaluated all the signaling pathways discussed in this review within the same experimental model. Tasinov et al. (2021) [9] assessed NF-κB-associated inflammatory markers and ER stress markers together in the same macrophage system, while all remaining mechanistic connections are based on converging evidence from independent studies of *S. ebulus* extracts and their constituent phytochemicals. The integrated synthesis presented below and in Figure 1 should be understood in this context.

The biological activity reviewed in the preceding sections is unlikely to arise from isolated single-target mechanisms. Rather, it reflects coordinated modulation of interconnected signaling networks involved in oxidative stress, inflammation, immune regulation, microbial defense, and tumorigenesis. This pattern characteristic of polyphenol-rich botanical preparations that may represent a genuine advantage in complex, multi-pathway diseases [54,115]. A schematic depicting these pathways, the crosstalk between them, and the sites of phytochemical action is presented in Figure 1.

Among the consistently implicated mechanisms, NF-κB suppression stands out as the most directly evidenced for *S. ebulus* fruit extracts specifically. Downstream reductions in multiple inflammatory markers have been documented at both transcriptomic and protein levels in macrophage models [9] and confirmed functionally in a human clinical study [10]. Nrf2 activation by the same phytochemicals acts as a counter-regulatory mechanism, upregulating antioxidant enzyme production, thereby reducing ROS accumulation and suppressing inflammatory amplification loops [9,74]. The mutual antagonism between NF-κB and Nrf2 is relevant in conditions characterized by persistent low-grade inflammation, including tumorigenesis, metabolic disorders, and cardiovascular disease [11,115].

MAPK, JAK/STAT, and PI3K/Akt pathways contribute additional mechanistic layers, inferred principally from studies of constituent phytochemicals in other systems. Suppression of ERK, JNK, and p38 phosphorylation by quercetin and anthocyanin derivatives reduces AP-1 activation and downstream inflammatory gene expression [57]; inhibition of STAT3 by resveratrol and quercetin limits expression of survivin, cyclin D1, Bcl-2, and VEGF [109]; and attenuation of Akt/mTOR signaling further reduces proliferation [70]. The convergence of these effects on shared downstream targets—Bcl-2 family proteins, MMPs, and pro-inflammatory cytokines—supports the view that *S. ebulus* phytochemicals act on overlapping nodes within an interconnected network rather than through independent parallel mechanisms.

The apoptosis-related mechanisms described in Section 6.3 are embedded within this same landscape. Quercetin and resveratrol shift the Bax/Bcl-2 ratio toward apoptosis and disrupt mitochondrial membrane potential, especially in tumor cells where elevated basal ROS increases vulnerability to polyphenol-mediated redox perturbation [73,108]. Direct *S. ebulus* evidence shows upregulation of Bax, Bak, and p53 in MDA-MB-231 cells with selectivity toward malignant versus normal breast cells [20], and modulation of apoptosis-related signaling in the AOM/DSS colon cancer model [19] and provides support for this mechanism, though mechanistic depth remains limited.

### 7.1. Future Perspectives for Unexplored Relevant Interactions

Several mechanistic dimensions of *S. ebulus* fruit activity remain insufficiently characterized and represent open avenues for future investigation. Endoplasmic reticulum (ER) stress modulation is among the best-supported of these. Chronic ER stress activates the unfolded protein response (UPR) via IRE1α, PERK, and ATF6, which when unresolved promotes NF-κB and MAPK activation and inflammatory cytokine production [25]. Tasinov et al. demonstrated that aqueous *S. ebulus* fruit extract reduced ER stress markers (p-eIF2α, ATF6, CHOP) in LPS-stimulated macrophages [9], and a subsequent review has argued that constituent phytochemicals (chlorogenic acid, epicatechin, resveratrol, chrysanthemin) reduce ER stress markers at low concentrations while high-dose resveratrol activates pro-apoptotic UPR signaling selectively in cancer cells [25], which warrants further investigation.

Autophagy regulation is another plausible but undemonstrated mechanism for dwarf elder activity. Constituent phytochemicals have the ability to modulate autophagy through converging mechanisms—resveratrol inhibits mTORC1 in an ATP-competitive manner and activates AMPK and SIRT1 [71,116], while quercetin induces autophagic flux alongside apoptosis via the p-STAT3/Bcl-2 axis [117]—and autophagy facilitates clearance of pro-inflammatory aggregates and NLRP3 inflammasome substrates [118]. Whether these mechanisms operate under *S. ebulus* fruit extract exposure has not been investigated.

Extracellular vesicle (EV) biology is similarly unexplored. Resveratrol and quercetin modulate exosome secretion and miRNA cargo in cancer cell lines [119,120], and polyphenols broadly influence EV biogenesis [121], but no evidence exists for *S. ebulus* extracts specifically.

Beyond these three mechanisms, several further directions merit attention. The interaction between dwarf elderberry phytochemicals and the gut microbiome, which governs polyphenol biotransformation and is likely a major determinant of in vivo activity, has received no dedicated study. The potential for *S. ebulus* polyphenols to modulate epigenetic regulators beyond miRNAs, including DNA methyltransferases and histone deacetylases, remains unexamined. Combination effects between dwarf elder preparations and conventional chemotherapeutic or anti-inflammatory agents have not been formally evaluated, despite the theoretical relevance of polyphenol-drug interactions. Finally, the development and characterization of delivery systems to overcome the bioavailability constraints discussed below, such as encapsulation or nanoformulation of standardized extracts, represents a key translational aspect.

### 7.2. Phytochemical Synergy, Bioavailability, and Standardization Considerations

Crude or partially purified dwarf elder extracts frequently exhibit stronger biological activity than equivalent concentrations of isolated individual compounds, a pattern that recurs across antimicrobial and tumor-suppressive endpoints and is consistent with additive or synergistic interactions, although formal synergy analysis using combination index or isobologram methods has not been performed for *S. ebulus* preparations [9,26]. Anthocyanins likely contribute primarily to antioxidant and anti-inflammatory effects; quercetin derivatives more strongly influence NF-κB and STAT signaling; chlorogenic acid supports redox regulation and metabolic modulation; while resveratrol modulates apoptosis, autophagy, and epigenetic regulation. Within the intact phytochemical matrix, these compounds appear to function cooperatively through complementary multi-target engagement [54].

Bioavailability is the most fundamental constraint on the translational relevance of the in vitro data reviewed here, and it warrants direct quantitative treatment. The concentrations used in *S. ebulus* cell studies are commonly 10–500 µg/mL for crude extracts and 10–100 µM for isolated compounds and are rarely achievable in human plasma after oral consumption of fruit preparations. For anthocyanins, the most abundant polyphenol class in the fruits, peak plasma concentrations following physiological intake are typically in the low nanomolar range (1–100 nM), with rapid clearance and extensive phase II conjugation [104]. Quercetin metabolites reach low micromolar concentrations at best, and resveratrol exhibits oral bioavailability of roughly 1% owing to rapid glucuronidation and sulfation [104]. No pharmacokinetic study has been performed for any *S. ebulus* fruit preparation in humans or animals, so there is no species-specific data to bridge in vitro concentrations and achievable tissue exposure. This gap is not a minor caveat: most of the mechanistic data reviewed in Section 4 and Section 6 were generated at concentrations that cannot be assumed to occur in target tissues after normal consumption.

The mechanisms governing this gap are compound-dependent. Lipophilic aglycones such as resveratrol and quercetin cross lipid bilayers by passive diffusion, whereas the glycosylated forms that predominate in the intact fruit require prior deglycosylation by intestinal brush border or microbiome enzymes before absorption [104]. Anthocyanins and flavonol glycosides additionally depend on transporter-mediated uptake via SGLT1 and GLUT2, making their absorption rate-limited and highly variable between individuals [21,72]. A substantial fraction of ingested anthocyanins and essentially all proanthocyanidins reach the colon intact and undergo extensive microbial biotransformation into lower-molecular-weight phenolic metabolites with potentially altered activity, an undercharacterized determinant of in vivo efficacy [49,104]. The gastrointestinal tract is therefore the most plausible site of physiologically relevant action for orally administered preparations, consistent with the colorectal model discussed in Section 6.2 [19]; systemic effects are harder to reconcile with achievable plasma levels.

Repeated low-level exposure to phytochemicals and their microbial metabolites may still modulate signaling pathways over prolonged periods, but pharmacokinetic characterization remains essential before translational conclusions can be drawn [21,104]. Substantial variability in extraction methods, geographic origin, fruit maturity, and phytochemical quantification further complicates inter-study comparison and standardization [26].

From a practical standpoint, the most relevant *S. ebulus* preparation documented in the literature is the aqueous fruit infusion—the traditional preparation used in Bulgaria and the Balkans and the format employed in both human intervention studies [10,11]. This format is accessible, thermally processed (reducing toxic constituent risk), and amenable to standardization through controlled drying and infusion conditions. The available human data suggest that regular consumption produces measurable anti-inflammatory and antioxidant effects at the systemic level [10,11], providing a plausible basis for further development as a functional food ingredient or standardized nutraceutical. Anthocyanin stability under processing and storage conditions, as discussed in Section 3, remains a key technical challenge for any such development.

### 7.3. Safety

Although properly processed ripe *S. ebulus* fruits appear considerably safer than other tissues of the plant, the toxicological profile of the species warrants more detailed discussion than is typically found in the literature. Ribosome-inactivating proteins (RIPs), including ebulin 1, are predominantly localized in leaves, stems, and rhizomes [52], and their concentrations in ripe fruit preparations are substantially lower, though complete absence across all cultivars and maturity stages has not been confirmed. Cyanogenic glycosides, including sambunigrin, are present at substantially higher concentrations in unripe material and other tissues than in ripe fruits; thermal processing through boiling or infusion significantly reduces cyanogenic compound levels through hydrolysis and volatilization of hydrogen cyanide [52], which is directly relevant to the safety of traditional infusion-based preparations. Lectins are similarly concentrated in non-fruit tissues, and ripe fruit preparations at typical dietary doses are not expected to contain toxic lectin concentrations, though dose-dependent effects have not been formally characterized. No formal acute toxicity, chronic toxicity, or genotoxicity studies such as Ames test or micronucleus assay are available specifically for ripe *S. ebulus* fruit preparations to our knowledge, representing a critical gap that must be addressed before any clinical or nutraceutical application can be recommended. Fruit maturity is therefore not *just* a phytochemical variable but a primary safety determinant, and standardization of ripeness criteria alongside validated processing protocols is an essential prerequisite for the development of safe, reproducible dwarf elderberry preparations.

### 7.4. Conclusions and Current Limitations

Dwarf elderberries represent a biologically active phytochemical matrix capable of engaging multiple interconnected pathways involved in oxidative stress, inflammation, microbial defense, and tumorigenesis. However, progress is constrained due to over-reliance on in vitro systems using crude, variably characterized extracts, insufficient pharmacokinetic characterization, and a near-complete absence of clinical investigations. Concentrations required to elicit biological effects in vitro may not be achievable in relevant tissues following oral administration, given well-documented limitations on polyphenol bioavailability [104], and the influence of the gut microbiome on polyphenol biotransformation and systemic bioactivity—likely a major determinant of in vivo efficacy—has received essentially no attention in the context of *S. ebulus*. Substantial heterogeneity in extraction methods, phytochemical profiles, and experimental models further limits cross-study comparison and reproducibility. Future work should address the following priorities:

- Standardized extract preparation with full phytochemical characterization and defined ripeness criteria;
- Mechanistic validation in well-powered in vivo models with dose–response data;
- Pharmacokinetic studies measuring polyphenol absorption, tissue distribution, and metabolite identification following food-relevant preparations;
- Formal genotoxicity, acute toxicity, and chronic toxicity assessment for ripe fruit preparations;
- Investigation of gut microbiome-dependent polyphenol biotransformation and its influence on systemic bioactivity;
- Evaluation of anthocyanin stability under food-relevant processing and storage conditions;
- Clinical pilot studies, with the anti-inflammatory endpoint representing the most mature area given existing human data [10,11];
- Assessment of potential interactions with co-administered drugs, particularly anticoagulants and anti-inflammatory agents, given the known effects of polyphenols on CYP450 enzymes and drug transporters.

Until these gaps are properly addressed, translational conclusions cannot be adequately drawn.

## Figures and Tables

**Table 1:** Phytochemical constituents identified and quantified in *Sambucus ebulus* L. fruit preparations by extraction method.

Compound Class	Compound	Concentration (as Reported)	Extraction Method	Analytical Method	Ref.
**AQUEOUS EXTRACT/INFUSION**					
Anthocyanins	Cyanidin-3-O-galactoside	48.15 mg/g DW; 382.15 µg/mL	Aqueous infusion; aqueous extract (PBS, pH 7.4)	UPLC-ESI-MS/MS; LC-MS/MS	[9,10]
	Cyanidin-3-O-sambubioside	43.41 ± 1.07 mg/g DW	Aqueous infusion	UPLC-ESI-MS/MS	[10]
	Cyanidin-3-O-arabinoside	10.82 mg/g DW	Aqueous infusion	UPLC-ESI-MS/MS	[10]
	Cyanidin-3-O-glucoside	Detected; dominant in some preparations	Aqueous extract	LC-MS/MS	[9]
	Cyanidin-3-O-xyloside	1.81 mg/g DW	Aqueous infusion	UPLC-ESI-MS/MS	[10]
	Total anthocyanins	1966.76 µg/mL	Aqueous extract	LC-MS/MS	[9]
Hydroxycinnamic acids	5-Caffeoylquinic acid (chlorogenic acid)	114.17 mg/g DW	Aqueous infusion	UPLC-DAD-ESI-MS/MS	[26]
	3-p-Coumaroylquinic acid	50.33 mg/g DW	Aqueous infusion	UPLC-DAD-ESI-MS/MS	[26]
	3-p-Feruloylquinic acid	31.36 mg/g DW	Aqueous infusion	UPLC-DAD-ESI-MS/MS	[26]
	p-Coumaric acid glucoside	29.78 mg/g DW	Aqueous infusion	UPLC-DAD-ESI-MS/MS	[26]
Flavonols	Quercetin-3-O-galactoside	3.68 mg/g DW	Aqueous infusion	UPLC-DAD-ESI-MS/MS	[26]
	Quercetin-3-O-rhamnosyl-galactoside	3.22 mg/g DW	Aqueous infusion	UPLC-DAD-ESI-MS/MS	[26]
	Quercetin-3-O-glucoside	2.87 mg/g DW	Aqueous infusion	UPLC-DAD-ESI-MS/MS	[26]
Proanthocyanidins/flavanols	Epicatechin	322.37 µg/mL	Aqueous extract	LC-MS/MS	[9]
	Catechin, proanthocyanidin dimers/trimers	Considerable amounts	Aqueous infusion	UPLC-DAD-ESI-MS/MS	[26]
Stilbenes	Resveratrol-3-O-glucoside (*trans*-piceid)	51.93 µg/mL	Aqueous extract	LC-MS/MS	[9]
	Resveratrol (free)	Exceeds red grape skin content †	Aqueous infusion	UPLC-DAD-ESI-MS/MS	[26]
Organic acids	Quinic acid, citric acid, malic acid	Detected; quinic acid predominant	Aqueous extract	GC-MS	[9]
Amino acids	Phenylalanine	10.25 µg/mL	Aqueous extract	GC-MS	[9]
	Isoleucine	8.48 µg/mL	Aqueous extract	GC-MS	[9]
	Leucine	8.06 µg/mL	Aqueous extract	GC-MS	[9]
**AQUEOUS-ETHANOLIC EXTRACTS (20–80% ethanol)**					
Total polyphenols	Total polyphenol content	Higher than aqueous alone; optimum at 40–60% ethanol	Aqueous-ethanolic (20%, 40%, 60%, 80% *v*/*v*)	Folin–Ciocalteu	[27]
Total anthocyanins	Total anthocyanins	Higher than aqueous alone; optimum at 20–40% ethanol	Aqueous-ethanolic (20%, 40%, 60%, 80% *v*/*v*)	pH-differential method	[27]
**HYDROALCOHOLIC/METHANOL EXTRACT**					
Hydroxycinnamic acids	Chlorogenic acid (5-CQA)	139.09 mg/g ext.	Hydroalcoholic (methanol:water)	LC-PDA-MS	[28]
	Sinapic acid	72.84 mg/g ext.	Hydroalcoholic	LC-PDA-MS	[28]
	*trans*-Cinnamic acid	51.29 mg/g ext.	Hydroalcoholic	LC-PDA-MS	[28]
Flavonols	Rutin (quercetin-3-O-rutinoside)	1105.39 mg/g ext. ‡	Hydroalcoholic	LC-PDA-MS	[28]
	Quercetin	306.6 mg/g ext. ‡	Hydroalcoholic	LC-PDA-MS	[28]
	Quercetin-3-rutinoside, kaempferol-3-rutinoside, isorhamnetin-3-rutinoside	Present as major flavonol glycosides	70% methanol	HPLC-MS	[29]
	Kaempferol glycosides (7 identified)	Present	Hydroalcoholic	HPLC-MS	[30]
	Isorhamnetin glycosides (8 identified)	Present	Hydroalcoholic	HPLC-MS	[30]
Total polyphenols	Total phenolic content	92.777 mg GAE/g	Methanol	Folin–Ciocalteu	[28]
**ETHYL ACETATE FRACTION**					
Hydroxycinnamic acids	Chlorogenic acid	Dominant non-anthocyanin phenolic; isolated as anti-inflammatory principle	Sequential: MeOH → EtOAc partition	HPLC; structure elucidation	[31]
Flavonols	Quercetin derivatives, kaempferol derivatives	Present as major flavonols in EtOAc fraction	Sequential: MeOH → EtOAc partition	HPLC-MS	[31]
**ACETONE EXTRACT/HYDROPHILIC AND ANTHOCYANIN-RICH FRACTIONS**					
Total polyphenols/anthocyanins	Enriched anthocyanin fraction (C); enriched phenolic acid/flavonol fraction (B); polar fraction (A)	High antioxidant activity; cytoprotective against tBHP	SPE fractionation of aqueous extract: fraction A (polar), B (EtOAc-eluted), C (anthocyanin, ACN/formic acid)	DPPH, tBHP cytotoxicity assay	[9]
**SUBCRITICAL WATER EXTRACT**					
Total polyphenols	Total phenolics, flavonoids	Varies with temperature (100–200 °C); higher polyphenol yield at 150 °C	Subcritical water (pressurized hot water, 100–200 °C)	HPLC; DPPH; ABTS	[32]

DW: dry weight; ext.: dried extract; GAE: gallic acid equivalents; UPLC-ESI-MS/MS: ultra-performance liquid chromatography with electrospray ionization tandem mass spectrometry; UPLC-DAD-ESI-MS/MS: ultra-performance liquid chromatography with diode array detection and electrospray ionization tandem mass spectrometry; LC-PDA-MS: liquid chromatography with photodiode array detection and mass spectrometry; HPLC-MS: high-performance liquid chromatography–mass spectrometry; HPLC: high-performance liquid chromatography; SPE: solid-phase extraction; DPPH: 2,2-diphenyl-1-picrylhydrazyl; ABTS: 2,2′-azino-bis(3-ethylbenzothiazoline-6-sulfonic acid). † Exact quantitative data for free resveratrol not reported in Kiselova-Kaneva et al. (2022) [26]; qualitative comparison with red grape skin stated by the authors. ‡ Values from Kayıran et al. (2022) [28] are expressed per gram of dried hydroalcoholic extract. These anomalously high values likely reflect concentration during extract preparation and are not directly comparable with mg/g DW values from aqueous infusion studies.

**Table 2:** Summary of direct immunomodulatory and anti-inflammatory findings from *Sambucus ebulus* L. fruit extract studies.

Extract Type	Model	Dose/Concentration	Key Endpoints	Key Findings	Ref.
Aqueous fruit extract (PBS, pH 7.4); SPE fractions A, B, C	LPS-stimulated J774A.1 macrophages; in vitro	10, 25, 50 µg/mL	mRNA expression of IL-1β, IL-6, TNF-α, COX-2, iNOS, ICAM-1; iNOS protein (Western blot); ER stress markers (p-eIF2α, ATF6α, CHOP)	Significant suppression of all six inflammatory gene transcripts; iNOS protein reduction confirmed; ER stress markers reduced; effects comparable to salicylic acid	[9]
Aqueous fruit infusion (traditional preparation)	53 healthy human volunteers; 4-week intervention	Standard infusion preparation	Serum IL-6, TNF-α, IL-8 (ELISA); complement system activity (C3, C4, CH50)	Significant reductions in IL-6 (20.15%), TNF-α (5.38%), IL-8 (5.50%); complement pathway modulation	[10]
Aqueous fruit infusion	22 healthy human volunteers; 4-week intervention	Standard infusion preparation	Serum antioxidant capacity (FRAP, DPPH); lipid profile (TC, TG, LDL, HDL)	Increased total antioxidant capacity; reduced serum triglycerides and total cholesterol	[11]
Ethanolic fruit extract	AOM/DSS colitis-associated colon cancer mouse model; in vivo	100 mg/kg/day, 14 days	Oxidative stress markers (MDA, SOD, CAT, GSH); apoptosis markers; TRP channel activity	Reduced oxidative stress; modulated apoptotic signaling; reduced TRP channel-mediated ROS	[19]
Hydroalcoholic fruit extract (70% ethanol)	Human nasal polyp tissue; ex vivo	50, 315, 1000 µg/mL; 24 h	IL-5, GM-CSF (ELISA); apoptosis (TUNEL); Bax, Bad mRNA (RT-PCR)	Significantly reduced GM-CSF; increased apoptosis and Bax/Bad expression in eosinophilic inflammatory cells; IL-5 not significantly changed	[55]

ELISA: enzyme-linked immunosorbent assay; RT-PCR: reverse transcription polymerase chain reaction; TUNEL: terminal deoxynucleotidyl transferase dUTP nick end labeling; SPE: solid-phase extraction; FRAP: ferric reducing antioxidant power; DPPH: 2,2-diphenyl-1-picrylhydrazyl; TC: total cholesterol; TG: triglycerides; LDL: low-density lipoprotein; HDL: high-density lipoprotein; MDA: malondialdehyde; GM-CSF: granulocyte-macrophage colony-stimulating factor. Note that Refs. [11,19] primarily report antioxidant/lipid and anticancer endpoints, respectively; they are included here as they provide direct evidence of anti-inflammatory or redox-modulating activity of *S. ebulus* fruit preparations in physiologically relevant models.

**Table 3:** Summary of antimicrobial findings from *Sambucus ebulus* L. fruit extract studies.

Extract Type	Target Organism	Assay Method	Key Finding/Effect	Ref.
Hydroalcoholic fruit extract	*Herpes simplex virus type 1* (HSV-1); Vero cell line	Plaque reduction assay (TCID_50_); quantitative RT-PCR; immunofluorescence (IFA) for HSV-1 antigen	2.6 log_10_ TCID_50_ reduction in viral titre; 91.2% inhibition of replication at 75 µg/mL (highest non-toxic concentration); HSV-1 antigen expression significantly reduced	[14]
Purified *S. ebulus* fruit extract vs. *S. nigra*	*Herpes simplex virus type 2* (HSV-2); MDBK cell line	Virucidal activity assay; HPLC polyphenol profiling	*S. ebulus* purified berry extract showed no significant HSV-2 virucidal activity; strong activity observed for *S. nigra* preparations; attributed to lower anthocyanin and phenolic acid content in *S. ebulus* fruit extract	[85]
70% methanol extract of *S. ebulus* fruit; SPE-purified flavonoid glycoside fractions	*Herpes simplex virus type 1* (HSV-1); Vero cell line	Cytopathic effect inhibition assay; HPLC-MS flavonoid glycoside profiling	Flavonoid glycoside fractions demonstrated anti-HSV-1 activity; quercetin-3-rutinoside and isorhamnetin-3-rutinoside identified as major active constituents; activity correlated with flavonoid glycoside content	[29]
Methanolic fruit extract	*Staphylococcus aureus* ATCC reference strain; 16 clinical MRSA isolates	Broth microdilution MIC; disc diffusion	All 16 clinical MRSA isolates inhibited; MIC 15 mg/mL against *S. aureus* ATCC reference strain	[15]
Ethanol fruit extract	*Bacillus subtilis*, *Enterococcus faecalis*, *Bacillus cereus*, *Staphylococcus aureus*, *Pseudomonas fluorescens*, *Escherichia coli* (bacteria); *Botrytis cinerea*, *Rhizoctonia solani*, *Phytophthora infestans* (fungi)	Disc diffusion (antibacterial); mycelial growth inhibition assay (antifungal); Folin–Ciocalteu total phenolics; DPPH antioxidant capacity	Activity against most tested strains; best antibacterial results against *P. fluorescens* and *E. faecalis*; antifungal inhibition of mycelial growth in all three fungal pathogens tested; activity correlated with total phenolic and flavonoid content	[86]
Dried fruit methanol extract; fresh fruit juice	*Escherichia coli*, *Proteus mirabilis*, *Staphylococcus aureus*, *Candida tropicalis*, *Candida albicans*, *Candida parapsilosis*, *Staphylococcus epidermidis*, *Trichophyton rubrum*	Broth microdilution MIC; LC-PDA-MS phytochemical characterization	Moderate antibacterial activity against *E. coli*, *P. mirabilis*, *S. aureus*; antifungal activity against *C. tropicalis* (MIC 312.5 mg/L); *C. albicans*, *C. parapsilosis*, *S. epidermidis*, and *T. rubrum* resistant to all preparations tested	[28]
Ethanolic fruit extract; GC/MS characterisation	*Saprolegnia parasitica* (fish pathogenic oomycete); in vitro	Hyphal growth inhibition assay; MIC determination	Complete inhibition of hyphal growth at ≥5% extract concentration; major constituents identified as fatty acids and phytol alongside polyphenolics	[87]
Fruit extract (extraction solvent not specified in abstract; likely aqueous-methanolic)	*Giardia lamblia* cysts; in vitro	Cytotoxicity against *G. lamblia* cysts isolated from patients; viability counting	Significant anti-giardial cytotoxicity against *G. lamblia* cysts at tested concentrations; *S. ebulus* fruit identified as a candidate natural antigiardial agent	[88]
Fruit extract (aqueous-methanolic)	*Echinococcus granulosus* protoscoleces (hydatid cyst); in vitro	Scolicidal activity assay; trypan blue viability staining at multiple time points and concentrations	Significant scolicidal activity against protoscoleces of *E. granulosus*; effect was concentration- and time-dependent; *S. ebulus* identified as a candidate scolicidal agent	[89]
Aqueous and ethanolic leaf and fruit extracts (comparative)	*Leishmania major* promastigotes and amastigotes; in vitro	MTT cytotoxicity; IC_50_ determination	Fruit extract showed significantly weaker antiparasitic activity than leaf extract against both stages; IC_50_ values for fruit extract considerably higher than for leaf preparations; results primarily attributable to leaf fraction	[90]
Leaf extract; in vivo mouse model (primarily leaf)	*Leishmania major*; cutaneous leishmaniasis mouse model	Lesion size measurement; IFN-γ and NO production; flow cytometry	Leaf extract stimulated cellular immune responses in vivo; fruit extract data limited; results primarily attributable to leaf fraction	[16]
Silver nanoparticles synthesized using *S. ebulus* fruit extract	*Toxoplasma gondii* tachyzoites; in vitro and in vivo mouse model	MTT assay; parasite burden quantification; histopathology	Significant antiparasitic activity in vitro and in vivo; note: effects cannot be attributed to fruit phytochemicals alone as silver nanoparticles independently possess antimicrobial activity and the contribution of each component was not isolated	[91]

MIC: minimum inhibitory concentration; TCID_50_: tissue culture infectious dose 50%; MRSA: methicillin-resistant Staphylococcus aureus; HPLC: high-performance liquid chromatography; LC-PDA-MS: liquid chromatography with photodiode array and mass spectrometric detection; GC/MS: gas chromatography/mass spectrometry; RT-PCR: reverse transcription polymerase chain reaction; IFA: indirect immunofluorescence assay; SPE: solid-phase extraction. Refs. [16,90] are included for completeness but primarily concern leaf preparations; findings should not be attributed to fruit extracts. Ref. [91] concerns silver nanoparticles synthesized using fruit extract; attribution of antimicrobial effects to phytochemical content alone is not possible from the available data.

## Data Availability

No new data were created or analyzed in this study. Data sharing is not applicable to this article.

## Abbreviations

The following abbreviations are used in this manuscript:

AOM/DSS	Azoxymethane/dextran sulfate sodium
AP-1	Activator protein-1
AMPK	AMP-activated protein kinase
ARE	Antioxidant response element
ATF6	Activating transcription factor 6
Akt	Protein kinase B
Bak	Bcl-2 homologous antagonist/killer
Bax	Bcl-2-associated X protein
Bcl-2	B-cell lymphoma 2
CAT	Catalase
CBP/p300	CREB-binding protein/E1A-binding protein p300
CHOP	C/EBP homologous protein
COX-2	Cyclooxygenase-2
EMT	Epithelial-to-mesenchymal transition
ER	Endoplasmic reticulum
ERK1/2	Extracellular signal-regulated kinases 1/2
EV	Extracellular vesicle
GC/MS	Gas chromatography/mass spectrometry
GPx	Glutathione peroxidase
HDAC3	Histone deacetylase 3
HO-1	Heme oxygenase-1
HSV-1	Herpes simplex virus type 1
HSV-2	Herpes simplex virus type 2
ICAM-1	Intercellular adhesion molecule-1
IFN	Interferon
IKK	IκB kinase
IL-1β	Interleukin-1 beta
IL-6	Interleukin-6
IL-8	Interleukin-8
iNOS	Inducible nitric oxide synthase
IRE1α	Inositol-requiring enzyme 1 alpha
IκBα	Inhibitor of kappa B alpha
JAK/STAT	Janus kinase/signal transducer and activator of transcription
JNK	c-Jun N-terminal kinase
Keap1	Kelch-like ECH-associated protein 1
LC-MS/MS	Liquid chromatography–tandem mass spectrometry
lncRNA	Long non-coding RNA
LPS	Lipopolysaccharide
MAPK	Mitogen-activated protein kinase
MIC	Minimum inhibitory concentration
miRNA	MicroRNA
MMP	Matrix metalloproteinase
mTOR	Mechanistic target of rapamycin
mTORC1	mTOR complex 1
MRSA	Methicillin-resistant Staphylococcus aureus
NADPH	Nicotinamide adenine dinucleotide phosphate
NF-κB	Nuclear factor kappa B
NLRP3	NLR family pyrin domain containing 3
NO	Nitric oxide
NQO1	NAD(P)H quinone oxidoreductase 1
Nrf2	Nuclear factor erythroid 2-related factor 2
p38	p38 mitogen-activated protein kinase
p53	Tumor protein p53
PAMP	Pathogen-associated molecular pattern
PERK	Protein kinase R-like ER kinase
PI3K	Phosphoinositide 3-kinase
RIP	Ribosome-inactivating protein
ROS	Reactive oxygen species
SIRT1	Sirtuin 1
SOD	Superoxide dismutase
STAT3	Signal transducer and activator of transcription 3
TGF-β	Transforming growth factor beta
TLR	Toll-like receptor
TNF-α	Tumor necrosis factor alpha
TRP	Transient receptor potential
ULK1	Unc-51-like autophagy activating kinase 1
UPR	Unfolded protein response
VCAM-1	Vascular cell adhesion molecule-1
VEGF	Vascular endothelial growth factor

## References

[1] Song M., Zhu X., Zhao X., Feng J., Sui X. (2026). Plant-Derived Bioactive Compounds in Inflammation-Related Cancers: Mechanisms and Therapeutic Potential. Plants.

[2] Wu Y., Antony S., Meitzler J.L., Doroshow J.H. (2014). Molecular Mechanisms Underlying Chronic Inflammation-Associated Cancers. Cancer Lett..

[3] Mitchell S., Vargas J., Hoffmann A. (2016). Signaling via the NFκB System. Wiley Interdiscip. Rev. Syst. Biol. Med..

[4] Philips R.L., Wang Y., Cheon H.J., Kanno Y., Gadina M., Sartorelli V., Horvath C.M., Darnell J.E., Stark G.R., O’Shea J.J. (2022). The JAK-STAT Pathway at 30: Much Learned, Much More to Do. Cell.

[5] Saha S., Buttari B., Panieri E., Profumo E., Saso L. (2020). An Overview of Nrf2 Signaling Pathway and Its Role in Inflammation. Molecules.

[6] Harnett J., Oakes K., Carè J., Leach M., Brown D., Cramer H., Pinder T.A., Steel A., Anheyer D. (2020). The Effects of *Sambucus nigra* Berry on Acute Respiratory Viral Infections: A Rapid Review of Clinical Studies. Adv. Integr. Med..

[7] Setz C., Rauch P., Setz M., Breitenberger S., Plattner S., Schubert U. (2025). Synergistic Antiviral Activity of European Black Elderberry Fruit Extract and Quinine Against SARS-CoV-2 and Influenza A Virusa. Nutrients.

[8] Rezaei-Moshaei M., Dehestani A., Bandehagh A., Pakdin-Parizi A., Golkar M., Heidari-Japelaghi R. (2021). Recombinant Pebulin Protein, a Type 2 Ribosome-Inactivating Protein Isolated from Dwarf Elder (*Sambucus ebulus* L.) Shows Anticancer and Antifungal Activities In Vitro. Int. J. Biol. Macromol..

[9] Tasinov O., Dincheva I., Badjakov I., Kiselova-Kaneva Y., Galunska B., Nogueiras R., Ivanova D. (2021). Phytochemical Composition, Anti-Inflammatory and ER Stress-Reducing Potential of *Sambucus ebulus* L. Fruit Extract. Plants.

[10] Kiselova-Kaneva Y., Nashar M., Roussev B., Salim A., Hristova M., Olczyk P., Komosinska-Vassev K., Dincheva I., Badjakov I., Galunska B. (2023). *Sambucus ebulus* (Elderberry) Fruits Modulate Inflammation and Complement System Activity in Humans. Int. J. Mol. Sci..

[11] Ivanova D., Tasinov O., Kiselova-Kaneva Y. (2014). Improved Lipid Profile and Increased Serum Antioxidant Capacity in Healthy Volunteers after *Sambucus ebulus* L. Fruit Infusion Consumption. Int. J. Food Sci. Nutr..

[12] Cassidy A., Rogers G., Peterson J.J., Dwyer J.T., Lin H., Jacques P.F. (2015). Higher Dietary Anthocyanin and Flavonol Intakes Are Associated with Anti-Inflammatory Effects in a Population of US Adults. Am. J. Clin. Nutr..

[13] Jurja S., Negreanu-Pirjol T., Mehedinți M.C., Hincu M.A., Negreanu-Pirjol B.S., Roncea F.N., Laurențiu Tatu A. (2025). Blueberries and Honeysuckle Berries: Anthocyanin-Rich Polyphenols for Vascular Endothelial Health and Cardiovascular Disease Prevention. Nutrients.

[14] Ghaffari H., Ataei-Pirkooh A., Mirghazanfari S.M., Barati M. (2021). Inhibition of Herpes Simplex Virus Type 1 Infection by *Sambucus ebulus* Extract In Vitro. Med. J. Islam. Repub. Iran..

[15] Salehzadeh A., Asadpour L., Naeemi A.S., Houshmand E. (2014). Antimicrobial Activity of Methanolic Extracts of *Sambucus ebulus* and *Urtica dioica* against Clinical Isolates of Methicillin Resistant *Staphylococcus aureus*. Afr. J. Tradit. Complement. Altern. Med..

[16] Heidari-Kharaji M., Fallah-Omrani V., Badirzadeh A., Mohammadi-Ghalehbin B., Nilforoushzadeh M.A., Masoori L., Montakhab-Yeganeh H., Zare M. (2019). *Sambucus ebulus* Extract Stimulates Cellular Responses in Cutaneous Leishmaniasis. Parasite Immunol..

[17] Ghosh S., Basu S., Anbarasu A., Ramaiah S. (2025). A Comprehensive Review of Antimicrobial Agents Against Clinically Important Bacterial Pathogens: Prospects for Phytochemicals. Phytother. Res..

[18] Zahmanova G., Takova K., Tonova V., Minkov I., Barbolov M., Nedeva N., Vankova D., Ivanova D., Kiselova-Kaneva Y., Lukov G.L. (2025). How Can Plant-Derived Natural Products and Plant Biotechnology Help Against Emerging Viruses?. Int. J. Mol. Sci..

[19] Kaya M.M., Kaya İ., Nazıroğlu M. (2023). Transient Receptor Potential Channel Stimulation Induced Oxidative Stress and Apoptosis in the Colon of Mice with Colitis-Associated Colon Cancer: Modulator Role of *Sambucus ebulus* L.. Mol. Biol. Rep..

[20] Omrani V.F., Koochaki A., Behzad S., Kia V., Ghasemi P., Razaviyan J., Moosavian H.R., Rezapour M., Vasei M., Asouri M. (2022). Effects of *Sambucus ebulus* Extract on Cell Proliferation and Viability of Triple-Negative Breast Cancer: An In Vitro and In Vivo Study. Anti-Cancer Agents Med. Chem.-Anti-Cancer Agents.

[21] Kumkum R., Aston-Mourney K., McNeill B.A., Hernández D., Rivera L.R. (2024). Bioavailability of Anthocyanins: Whole Foods versus Extracts. Nutrients.

[22] Jabbari M., Daneshfard B., Emami Alorizi S.M., Naseri M., Tabarrai M. (2017). Biological effects and clinical applications of dwarf elder (*Sambucus ebulus* L.): A review. J. Evid.-Based Complement. Altern. Med..

[23] Merecz-Sadowska A., Sitarek P., Zajdel K., Sztandera W., Zajdel R. (2024). Genus *Sambucus*: Exploring its potential as a functional food ingredient with neuroprotective properties mediated by antioxidant and anti-inflammatory mechanisms. Int. J. Mol. Sci..

[24] Ebadi A.G. (2025). Exploring the untapped therapeutic potential of *Sambucus ebulus*: Emerging phytochemical insights and medicinal applications. Biomed. J. Sci. Tech. Res..

[25] Stoyanov S., Barbolov M., Yaneva G., Tasinov O. (2025). Modulation of Endoplasmic Reticulum Stress by Selected Polyphenols from *Sambucus ebulus* L. Fruit. Plants.

[26] Kiselova-Kaneva Y., Galunska B., Nikolova M., Dincheva I., Badjakov I. (2022). High Resolution LC-MS/MS Characterization of Polyphenolic Composition and Evaluation of Antioxidant Activity of *Sambucus ebulus* Fruit Tea Traditionally Used in Bulgaria as a Functional Food. Food Chem..

[27] Tasinov O., Kiselova-Kaneva Y., Ivanova D. (2012). Antioxidant activity, total polyphenol content and anthocyanins content of *Sambucus ebulus* L. aqueous and aqueous-ethanolic extracts depend on the type and concentration of extragent. Sci. Technol..

[28] Kayıran S., Eroğlu Özkan E., Mataracı Kara E., Yazıcı Bektaş N., Tarı Ö., Nenni M. (2022). Determination of the Chemical Composition, Antioxidant Potential of *Sambucus ebulus* L. (Dwarf Elder) Fruit Extracts and Investigation of Antimicrobial Activity on Trichophyton Rubrum (Castell.) Sabour and Some Microorganisms. İStanbul J. Pharm..

[29] Zahmanov G., Alipieva K., Denev P., Todorov D., Hinkov A., Shishkov S., Simova S., Georgiev M.I. (2015). Flavonoid glycosides profiling in dwarf elder fruits (*Sambucus ebulus* L.) and evaluation of their antioxidant and anti-herpes simplex activities. Ind. Crops Prod..

[30] Mikulic-Petkovsek M., Ivancic A., Todorovic B., Veberic R., Stampar F. (2015). Fruit Phenolic Composition of Different Elderberry Species and Hybrids. J. Food Sci..

[31] Tasinov O.B., Kiselova-Kaneva Y.D., Nazifova-Tasinova N.F., Todorova M.N., Trendafilova A.B., Ivanova D.G. (2020). Chemical composition and cytoprotective and anti-inflammatory potential of *Sambucus ebulus* fruit ethyl acetate fraction. Bulg. Chem. Commun..

[32] Cvetanović A., Zeković Z., Švarc-Gajić J., Razić S., Damjanović A., Zengin G., Delerue-Matos C., Moreira M. (2018). A New Source for Developing Multi-Functional Products: Biological and Chemical Perspectives on Subcritical Water Extracts of *Sambucus ebulus* L.. J. Chem. Technol. Biotechnol..

[33] Jomova K., Alomar S.Y., Valko R., Liska J., Nepovimova E., Kuca K., Valko M. (2025). Flavonoids and Their Role in Oxidative Stress, Inflammation, and Human Diseases. Chem.-Biol. Interact..

[34] Ngamsamer C., Sirivarasai J., Sutjarit N. (2022). The Benefits of Anthocyanins against Obesity-Induced Inflammation. Biomolecules.

[35] Cavalcanti R.N., Santos D.T., Meireles M.A.A. (2011). Non-thermal stabilization mechanisms of anthocyanins in model and food systems—An overview. Food Res. Int..

[36] Castañeda-Ovando A., Pacheco-Hernández M.L., Páez-Hernández M.E., Rodríguez J.A., Galán-Vidal C.A. (2009). Chemical studies of anthocyanins: A review. Food Chem..

[37] Li X., Wang M., Pan S., Xian L., Zhang S., Xian D., Zhong J. (2024). Proanthocyanidins Alleviate Henoch-Schönlein Purpura by Mitigating Inflammation and Oxidative Stress through Regulation of the TLR4/MyD88/NF-ΚB Pathway. Skin. Res. Technol..

[38] Prokop A., Magiera A., Olszewska M.A. (2025). Proanthocyanidins as Therapeutic Agents in Inflammation-Related Skin Disorders. Int. J. Mol. Sci..

[39] Li Y., Yao J., Han C., Yang J., Chaudhry M.T., Wang S., Liu H., Yin Y. (2016). Quercetin, Inflammation and Immunity. Nutrients.

[40] Wu J., Lv T., Liu Y., Liu Y., Han Y., Liu X., Peng X., Tang F., Cai J. (2024). The Role of Quercetin in NLRP3-Associated Inflammation. Inflammopharmacology.

[41] Dong H., Song G., Wang Z., Wu X., Wang Q., Wang Y.H. (2025). Kaempferol as a Multifaceted Immunomodulator: Implications for Inflammation, Autoimmunity, and Cancer. Front. Immunol..

[42] Koli D., Sheokand A., Sharma G., Tuli H.S., Pahwa R. (2026). Harnessing Isorhamnetin for Gastrointestinal Cancers: Molecular Mechanisms and Therapeutic Potential. J. Biochem. Mol. Toxicol..

[43] Zhang L., Virgous C., Si H. (2019). Synergistic Anti-Inflammatory Effects and Mechanisms of Combined Phytochemicals. J. Nutr. Biochem..

[44] Ayaz M., Ullah F., Sadiq A., Ullah F., Ovais M., Ahmed J., Devkota H.P. (2019). Synergistic Interactions of Phytochemicals with Antimicrobial Agents: Potential Strategy to Counteract Drug Resistance. Chem.-Biol. Interact..

[45] Nguyen V., Taine E.G., Meng D., Cui T., Tan W. (2024). Chlorogenic Acid: A Systematic Review on the Biological Functions, Mechanistic Actions, and Therapeutic Potentials. Nutrients.

[46] Chen B., Wang L., Xie R., Li B., Peng S., Ou Y., Zhuang R., Zhuang W., Huang H., Wu J. (2025). Inflammation-Targeted and Antioxidative Poly(Ferulic Acid) Nanoparticles Directly Treat Chronic Nonbacterial Prostatitis via Inhibiting Pyroptosis by Disrupting Nrf2/KEAP1 Multimer Formation and as a Robust Drug Carrier. Adv. Healthc. Mater..

[47] Zhang J., Ouyang H., Gu X., Dong S., Lu B., Huang Z., Li J., Ji L. (2024). Caffeic Acid Ameliorates Metabolic Dysfunction-Associated Steatotic Liver Disease via Alleviating Oxidative Damage and Lipid Accumulation in Hepatocytes through Activating Nrf2 via Targeting Keap1. Free Radic. Biol. Med..

[48] Ghasemi-Dehnoo M., Lorigooini Z., Amini-Khoei H., Sabzevary-Ghahfarokhi M., Rafieian-Kopaei M. (2023). Quinic Acid Ameliorates Ulcerative Colitis in Rats, through the Inhibition of Two TLR4-NF-ΚB and NF-ΚB-INOS-NO Signaling Pathways. Immun. Inflamm. Dis..

[49] Li S., Cai Y., Guan T., Zhang Y., Huang K., Zhang Z., Cao W., Guan X. (2024). Quinic Acid Alleviates High-Fat Diet-Induced Neuroinflammation by Inhibiting DR3/IKK/NF-ΚB Signaling via Gut Microbial Tryptophan Metabolites. Gut Microbes.

[50] Meng T., Xiao D., Muhammed A., Deng J., Chen L., He J. (2021). Anti-Inflammatory Action and Mechanisms of Resveratrol. Molecules.

[51] Ren B., Kwah M.X.Y., Liu C., Ma Z., Shanmugam M.K., Ding L., Xiang X., Ho P.C.L., Wang L., Ong P.S. (2021). Resveratrol for Cancer Therapy: Challenges and Future Perspectives. Cancer Lett..

[52] Girbés T., Citores L., Iglesias R., Ferreras J.M., Muñoz R., Rojo M.A., Arias F.J., García J.R., Méndez E., Calonge M. (1993). Ebulin 1, a Nontoxic Novel Type 2 Ribosome-Inactivating Protein from *Sambucus ebulus* L. *Leaves*. J. Biol. Chem..

[53] Furman D., Campisi J., Verdin E., Carrera-Bastos P., Targ S., Franceschi C., Ferrucci L., Gilroy D.W., Fasano A., Miller G.W. (2019). Chronic Inflammation in the Etiology of Disease across the Life Span. Nat. Med..

[54] Williamson G. (2017). The Role of Polyphenols in Modern Nutrition. Nutr. Bull..

[55] Pourgholamali B., Yosefbeyk F., Ansar M.M., Zaminy A., Nemati S., Ramezani S., Bagheri H., Faghani M. (2024). Phytochemical content, anti-inflammatory, anti-apoptotic, and antioxidant activities of dwarf elder (*Sambucus ebulus*) against nasal polyposis. Jundishapur J. Nat. Pharm. Prod..

[56] Mao H., Zhao X., Sun S.C. (2025). NF-ΚB in Inflammation and Cancer. Cell. Mol. Immunol..

[57] Cho S.Y., Park S.J., Kwon M.J., Jeong T.S., Bok S.H., Choi W.Y., Jeong W.-I., Ryu S.Y., Do S.H., Lee C.S. (2003). Quercetin Suppresses Proinflammatory Cytokines Production through MAP Kinases AndNF-KappaB Pathway in Lipopolysaccharide-Stimulated Macrophage. Mol. Cell. Biochem..

[58] Kim E.K., Choi E.J. (2015). Compromised MAPK Signaling in Human Diseases: An Update. Arch. Toxicol..

[59] Xia M., Ling W., Zhu H., Ma J., Wang Q., Hou M., Tang Z., Guo H., Liu C., Ye Q. (2009). Anthocyanin Attenuates CD40-Mediated Endothelial Cell Activation and Apoptosis by Inhibiting CD40-Induced MAPK Activation. Atherosclerosis.

[60] Zhang S., Liang W., Abulizi Y., Xu T., Cao R., Xun C., Zhang J., Sheng W. (2021). Quercetin Alleviates Intervertebral Disc Degeneration by Modulating P38 MAPK-Mediated Autophagy. BioMed Res. Int..

[61] Ling X., Yan W., Yang F., Jiang S., Chen F., Li N. (2023). Research Progress of Chlorogenic Acid in Improving Inflammatory Diseases. J. Cent. South Univ. Med. Sci..

[62] Omraninava M., Razi B., Aslani S., Imani D., Jamialahmadi T., Sahebkar A. (2021). Effect of Resveratrol on Inflammatory Cytokines: A Meta-Analysis of Randomized Controlled Trials. Eur. J. Pharmacol..

[63] Pereira Q.C., dos Santos T.W., Fortunato I.M., Ribeiro M.L. (2023). The Molecular Mechanism of Polyphenols in the Regulation of Ageing Hallmarks. Int. J. Mol. Sci..

[64] Yu H., Pardoll D., Jove R. (2009). STATs in Cancer Inflammation and Immunity: A Leading Role for STAT3. Nat. Rev. Cancer.

[65] Kowalczyk T., Muskała M., Merecz-Sadowska A., Sikora J., Picot L., Sitarek P. (2024). Anti-Inflammatory and Anticancer Effects of Anthocyanins in In Vitro and In Vivo Studies. Antioxidants.

[66] Yin Q., Wang L., Yu H., Chen D., Zhu W., Sun C. (2021). Pharmacological Effects of Polyphenol Phytochemicals on the JAK-STAT Signaling Pathway. Front. Pharmacol..

[67] Zalpoor H., Nabi-Afjadi M., Forghaniesfidvajani R., Tavakol C., Farahighasreaboonasr F., Pakizeh F., Dana V.G., Seif F. (2022). Quercetin as a JAK-STAT Inhibitor: A Potential Role in Solid Tumors and Neurodegenerative Diseases. Cell. Mol. Biol. Lett..

[68] Roy T., Boateng S.T., Uddin M.B., Banang-Mbeumi S., Yadav R.K., Bock C.R., Folahan J.T., Siwe-Noundou X., Walker A.L., King J.A. (2023). The PI3K-Akt-MTOR and Associated Signaling Pathways as Molecular Drivers of Immune-Mediated Inflammatory Skin Diseases: Update on Therapeutic Strategy Using Natural and Synthetic Compounds. Cells.

[69] Wang W., Yuan X., Mu J., Zou Y., Xu L., Chen J., Zhu X., Li B., Zeng Z., Wu X. (2023). Quercetin Induces MGMT+ Glioblastoma Cells Apoptosis via Dual Inhibition of Wnt3a/β-Catenin and Akt/NF-ΚB Signaling Pathways. Phytomedicine.

[70] Unnikrishnan Meenakshi D., Narde G.K., Ahuja A., Al Balushi K., Francis A.P., Khan S.A. (2024). Therapeutic Applications of Nanoformulated Resveratrol and Quercetin Phytochemicals in Colorectal Cancer-An Updated Review. Pharmaceutics.

[71] Park D., Jeong H., Lee M.N., Koh A., Kwon O., Yang Y.R., Noh J., Suh P.G., Park H., Ryu S.H. (2016). Resveratrol Induces Autophagy by Directly Inhibiting MTOR through ATP Competition. Sci. Rep..

[72] Williamson G., Sheedy K. (2020). Effects of Polyphenols on Insulin Resistance. Nutrients.

[73] Reyes-Farias M., Carrasco-Pozo C. (2019). The Anti-Cancer Effect of Quercetin: Molecular Implications in Cancer Metabolism. Int. J. Mol. Sci..

[74] Wardyn J.D., Ponsford A.H., Sanderson C.M. (2015). Dissecting Molecular Cross-Talk between Nrf2 and NF-ΚB Response Pathways. Biochem. Soc. Trans..

[75] Ma Q. (2013). Role of Nrf2 in Oxidative Stress and Toxicity. Annu. Rev. Pharmacol. Toxicol..

[76] Liu X., Tan X., Su Y., Zou L., Yuan W., Yu C. (2025). Anthocyanin Attenuates Pulmonary Arterial Hypertension and Associated Heart Failure via Improving Mitochondrial Function through Nrf2-Dependent Mechanism. Clin. Exp. Hypertens..

[77] Peng C., Ai Q., Zhao F., Li H., Sun Y., Tang K., Yang Y., Chen N., Liu F. (2024). Quercetin Attenuates Cerebral Ischemic Injury by Inhibiting Ferroptosis via Nrf2/HO-1 Signaling Pathway. Eur. J. Pharmacol..

[78] Zheng Y., Li L., Chen B., Fang Y., Lin W., Zhang T., Feng X., Tao X., Wu Y., Fu X. (2022). Chlorogenic Acid Exerts Neuroprotective Effect against Hypoxia-Ischemia Brain Injury in Neonatal Rats by Activating Sirt1 to Regulate the Nrf2-NF-ΚB Signaling Pathway. Cell Commun. Signal..

[79] Pourbagher-Shahri A.M., Farkhondeh T., Jafari-Nozad A.M., Darroudi M., Naseri K., Amirian M., Samarghandian S. (2024). Nrf2 Mediates Effect of Resveratrol in Ischemia-Reperfusion Injury. Curr. Mol. Pharmacol..

[80] Rojo de la Vega M., Chapman E., Zhang D.D. (2018). NRF2 and the hallmarks of cancer. Cancer Cell.

[81] Menegon S., Columbano A., Giordano S. (2016). The dual roles of NRF2 in cancer. Trends Mol. Med..

[82] Mendelson M., Matsoso M.P. (2015). The World Health Organization Global Action Plan for Antimicrobial Resistance. S. Afr. Med. J..

[83] Sharma A., Anurag, Kaur J., Kesharwani A., Parihar V.K. (2024). Antimicrobial Potential of Polyphenols: An Update on Alternative for Combating Antimicrobial Resistance. Med. Chem..

[84] Suurbaar J., Mosobil R., Donkor A.M. (2017). Antibacterial and Antifungal Activities and Phytochemical Profile of Leaf Extract from Different Extractants of Ricinus Communis against Selected Pathogens. BMC Res. Notes.

[85] Seymenska D., Shishkova K., Hinkov A., Benbassat N., Teneva D., Denev P. (2023). Comparative Study on Phytochemical Composition, Antioxidant, and Anti-HSV-2 Activities of *Sambucus nigra* L. and *Sambucus ebulus* L. Extracts. Appl. Sci..

[86] Rodino S., Butu A., Petrache P., Butu M., Dinu-Pîrvu C.E., Cornea C.P. (2015). Evaluation of the antimicrobial and antioxidant activity of *Sambucus ebulus* extract. Farmacia.

[87] Mirmazloomi S., Ghiasi M., Khosravi A.R. (2022). Chemical Composition and In Vitro Antifungal Activity of *Sambucus ebulus* and *Actinidia deliciosa* on the Fish Pathogenic Fungus, *Saprolegnia parasitica*. Aquac. Int..

[88] Rahimi-Esboei B., Ebrahimzadeh M.A., Gholami S., Falah-Omrani V. (2013). Anti-giardial activity of *Sambucus ebulus*. Eur. Rev. Med. Pharmacol. Sci..

[89] Gholami S., Rahimi-Esboei B., Ebrahimzadeh M.A., Pourhajibagher M. (2013). In vitro effect of *Sambucus ebulus* on scolices of hydatid cysts. Eur. Rev. Med. Pharmacol. Sci..

[90] Kadkhodamasoum S., Bineshian F., KarimiPour A., Tavakoli P., Foroutan M., Ghaffarifar F., Molaei S. (2021). Comparison of the Effects of *Sambucus ebulus* Leaf and Fruit Extracts on Leishmania Major In Vitro. Infect. Disord. Drug Targets.

[91] Hematizadeh A., Ebrahimzadeh M.A., Sarvi S., Sadeghi M., Daryani A., Gholami S., Nayeri T., Hosseini S.A. (2023). In Vitro and In Vivo Anti-Parasitic Activity of *Sambucus ebulus* and Feijoa Sellowiana Extracts Silver Nanoparticles on Toxoplasma Gondii Tachyzoites. Acta Parasitol..

[92] Di Petrillo A., Orrù G., Fais A., Fantini M.C. (2022). Quercetin and Its Derivates as Antiviral Potentials: A Comprehensive Review. Phytother. Res..

[93] Roy A.V., Chan M., Banadyga L., He S., Zhu W., Chrétien M., Mbikay M. (2024). Quercetin Inhibits SARS-CoV-2 Infection and Prevents Syncytium Formation by Cells Co-Expressing the Viral Spike Protein and Human ACE2. Virol. J..

[94] Chen X., Song X., Zhao X., Zhang Y., Wang Y., Jia R., Zou Y., Li L., Yin Z. (2022). Insights into the Anti-Inflammatory and Antiviral Mechanisms of Resveratrol. Mediat. Inflamm..

[95] Wahedi H.M., Ahmad S., Abbasi S.W. (2021). Stilbene-Based Natural Compounds as Promising Drug Candidates against COVID-19. J. Biomol. Struct. Dyn..

[96] Burkard M., Piotrowsky A., Leischner C., Detert K., Venturelli S., Marongiu L. (2025). The Antiviral Activity of Polyphenols. Mol. Nutr. Food Res..

[97] Soleymani S., Zabihollahi R., Shahbazi S., Bolhassani A. (2018). Antiviral Effects of Saffron and Its Major Ingredients. Curr. Drug Deliv..

[98] Bouarab-Chibane L., Forquet V., Lantéri P., Clément Y., Léonard-Akkari L., Oulahal N., Degraeve P., Bordes C. (2019). Antibacterial Properties of Polyphenols: Characterization and QSAR (Quantitative Structure-Activity Relationship) Models. Front. Microbiol..

[99] Wu T., Zang X., He M., Pan S., Xu X. (2013). Structure-Activity Relationship of Flavonoids on Their Anti-Escherichia Coli Activity and Inhibition of DNA Gyrase. J. Agric. Food Chem..

[100] Tian L., Wu M., Guo W., Li H., Gai Z., Gong G. (2021). Evaluation of the Membrane Damage Mechanism of Chlorogenic Acid against Yersinia Enterocolitica and Enterobacter Sakazakii and Its Application in the Preservation of Raw Pork and Skim Milk. Molecules.

[101] Gyawali R., Ibrahim S.A. (2014). Natural Products as Antimicrobial Agents. Food Control.

[102] Tarahovsky Y.S. (2022). Hitchhiking into a Cell: Flavonoids May Produce Complexes with Transition Metals for Transmembrane Translocation. Biometals.

[103] Hanahan D. (2022). Hallmarks of Cancer: New Dimensions. Cancer Discov..

[104] Manach C., Williamson G., Morand C., Scalbert A., Rémésy C. (2005). Bioavailability and Bioefficacy of Polyphenols in Humans. I. Review of 97 Bioavailability Studies. Am. J. Clin. Nutr..

[105] Saravi S.S., Shokrzadeh M., Mirzayi M. (2009). Cytotoxic Effects of Ethyl Acetate Extract of *Sambucus ebulus* Compared with Etoposide on Normal and Cancer Cell Lines. Planta Med..

[106] León-González A.J., Auger C., Schini-Kerth V.B. (2015). Pro-Oxidant Activity of Polyphenols and Its Implication on Cancer Chemoprevention and Chemotherapy. Biochem. Pharmacol..

[107] Dzhalilova D., Zolotova N., Fokichev N., Makarova O. (2023). Murine Models of Colorectal Cancer: The Azoxymethane (AOM)/Dextran Sulfate Sodium (DSS) Model of Colitis-Associated Cancer. PeerJ.

[108] Imran M., Rauf A., Shah Z.A., Saeed F., Imran A., Arshad M.U., Ahmad B., Bawazeer S., Atif M., Peters D.G. (2019). Chemo-Preventive and Therapeutic Effect of the Dietary Flavonoid Kaempferol: A Comprehensive Review. Phytother. Res..

[109] Kursvietiene L., Kopustinskiene D.M., Staneviciene I., Mongirdiene A., Kubová K., Masteikova R., Bernatoniene J. (2023). Anti-Cancer Properties of Resveratrol: A Focus on Its Impact on Mitochondrial Functions. Antioxidants.

[110] Moslehi A., Komeili-Movahhed T., Ahmadian M., Ghoddoosi M., Heidari F. (2023). Chlorogenic Acid Attenuates Liver Apoptosis and Inflammation in Endoplasmic Reticulum Stress-Induced Mice. Iran. J. Basic Med. Sci..

[111] Cai Y., Li X., Pan Z., Zhu Y., Tuo J., Meng Q., Dai G., Yang G., Pan Y. (2020). Anthocyanin Ameliorates Hypoxia and Ischemia Induced Inflammation and Apoptosis by Increasing Autophagic Flux in SH-SY5Y Cells. Eur. J. Pharmacol..

[112] Huminiecki L. (2022). Evidence for Multilevel Chemopreventive Activities of Natural Phenols from Functional Genomic Studies of Curcumin, Resveratrol, Genistein, Quercetin, and Luteolin. Int. J. Mol. Sci..

[113] Shakeri A., Ghanbari M., Tasbandi A., Sahebkar A. (2021). Regulation of MicroRNA-21 Expression by Natural Products in Cancer. Phytother. Res..

[114] Kim D.H., Khan H., Ullah H., Hassan S.T.S., Šmejkal K., Efferth T., Mahomoodally M.F., Xu S., Habtemariam S., Filosa R. (2019). MicroRNA Targeting by Quercetin in Cancer Treatment and Chemoprotection. Pharmacol. Res..

[115] Alam M.N., Almoyad M., Huq F. (2018). Polyphenols in Colorectal Cancer: Current State of Knowledge Including Clinical Trials and Molecular Mechanism of Action. BioMed Res. Int..

[116] Filippi-Chiela E.C., Villodre E.S., Zamin L.L., Lenz G. (2011). Autophagy Interplay with Apoptosis and Cell Cycle Regulation in the Growth Inhibiting Effect of Resveratrol in Glioma Cells. PLoS ONE.

[117] Liu Y., Gong W., Yang Z.Y., Zhou X.S., Gong C., Zhang T.R., Wei X., Ma D., Ye F., Gao Q.L. (2017). Quercetin Induces Protective Autophagy and Apoptosis through ER Stress via the P-STAT3/Bcl-2 Axis in Ovarian Cancer. Apoptosis.

[118] Maiuri M.C., Kroemer G. (2019). Therapeutic Modulation of Autophagy: Which Disease Comes First?. Cell Death Differ..

[119] Tong K., Wang P., Li Y., Tong Y., Li X., Yan S., Hu P. (2024). Resveratrol Inhibits Hepatocellular Carcinoma Progression through Regulating Exosome Secretion. Curr. Med. Chem..

[120] Becer E., Özsoy S., Kabadayı H., Vatansever H.S. (2023). Quercetin change the exosome secretion and total miRNA concentration in primary (Colo320) and metastatic (Colo741) colon cancer cell lines. Bezmialem Sci..

[121] Soleti R., Andriantsitohaina R., Martinez M.C. (2018). Impact of Polyphenols on Extracellular Vesicle Levels and Effects and Their Properties as Tools for Drug Delivery for Nutrition and Health. Arch. Biochem. Biophys..

